# Influence of Genetic Variants in Type I Interferon Genes on Melanoma Survival and Therapy

**DOI:** 10.1371/journal.pone.0050692

**Published:** 2012-11-27

**Authors:** Romina Elizabeth Lenci, Melanie Bevier, Andreas Brandt, Justo Lorenzo Bermejo, Antje Sucker, Iris Moll, Dolores Planelles, Celia Requena, Eduardo Nagore, Kari Hemminki, Dirk Schadendorf, Rajiv Kumar

**Affiliations:** 1 Division of Molecular Genetic Epidemiology, German Cancer Research Center, Heidelberg, Germany; 2 Institute of Medical Biometry and Informatics, University of Heidelberg, Heidelberg, Germany; 3 Department of Dermatology, University Hospital Essen, Essen, Germany; 4 Department of Dermatology, Instituto Valenciano de Oncología, Valencia, Spain; College of Pharmacy, University of Florida, United States of America

## Abstract

Melanoma is an immunogenic tumor; however, the efficacy of immune-therapy shows large inter-individual variation with possible influence of background genetic variation. In this study we report the influence of genetic polymorphisms in the type I interferon gene cluster on chromosome 9p22 on melanoma survival. We genotyped 625 melanoma patients recruited in an oncology center in Germany for 44 polymorphisms located on chromosome 9p22 that were informative for 299 polymorphisms and spanned 15 type I interferon genes. Our results showed associations between time to metastasis/survival and two linked (r^2^ = 0.76) polymorphisms, rs10964859 (C>G) and rs10964862 (C>A). The rs10964859 polymorphism was located at 3′UTR and rs10964862 was 9.40 Kb towards 5′UTR of *IFNW1* gene. The carriers of the variant alleles of the rs10964859 and rs10964862 polymorphisms were associated with a reduced disease-free survival. The validation of data in an independent group of 710 patients from Spain showed that the direction of the effect was similar. Stratification based on therapy showed that the adverse effect on metastasis development was statistically significant in the patients from Spain who did not receive any treatment and were homozygous for variant allele of rs10964862 (HR = 2.52, 95% CI 1.07–5.90; P = 0.03). Patients homozygous for rs10964859 (HR = 2.01, 95% CI 1.17–3.44; P = 0.01) and rs10964862 (HR 1.84, 95%CI 1.03–3.27, P = 0.04) were associated to increased risk of death following metastasis. **G**TCG**A**CAA haplotype, found in 8.8% of the patients, was associated with an increased risk of death (HR 1.94, 95%CI 1.16–3.26, P = 0.01). In conclusion, our results identified genetic variants in interferon genes that influence melanoma progression and survival with modulation of effect due to treatment status.

## Introduction

Melanoma is considered a highly immunogenic tumor [1]–[3]. Strong evidence of the interaction of melanoma with the immune system has been supported by spontaneous remissions of growing melanomas [4], presence of tumor-infiltrated lymphocytes and development of vitiligo, which is associated with improved prognosis [5]. An increased incidence of melanoma tumors in transplant recipients [6] and evidence of immunoevasion are also common occurrences [7]. Immunotherapy has been one of the standard treatments in combating metastasized disease, however, the response rate remains poor [8], [9]. In order to increase humoral and cell mediated immunity against melanoma, therapies that include cytokines are used particularly in the adjuvant setting. Interferon-alpha (IFNA), a type I IFN has been reproducibly shown to affect melanoma behavior in humans and despite side effects it has been associated with prolonged relapse-free survival [10]–[13]. A clear effect on disease free and distant metastasis free survival but not on the overall survival was observed in high risk patients [14], [15]. Evidence suggested that the level of responsiveness to IFN treatment varies among individuals. Among the different possible causes for these inter-subject variations are the genetic polymorphisms. Associations of genetic variants in several type I *IFN* genes with different diseases, including melanoma have been reported in several studies [16]–[21].

Human type I *IFN* genes are located on chromosome 9p and, with an exception of *IFN kappa*, form a cluster upstream of the *CDKN2A* and *CDKN2B* tumor-suppressor genes and the noncoding antisense RNA encoded by *CDKN2BAS*. The region is frequently mutated and deleted in a wide variety of tumors and associated with melanoma [22], [23]. In this work, we aimed at surveying an entire set of variants spanning a 342 kb region with type I *IFN* genes with focus on the cluster on chromosome 9p22. The role of genetic variants in the type I *IFN* genes was investigated for association with melanoma survival and therapy in melanoma patients recruited in Germany. The results from the investigation of these polymorphisms were additionally confirmed in an independent group of patients recruited in Spain.

## Materials and Methods

### Study Population

The study was carried out on 752 German cutaneous melanoma patients. Survival analysis included 625 patients diagnosed at AJCC stage 0, I and II; complete data for age, gender and Breslow thickness were available for 541 patients (Table 1). Melanoma cases were referred to the Skin Cancer Unit, German Cancer Research Center Heidelberg, at the University Hospital Mannheim. For validation purposes, 837 patients from Spain were added to the study, which included 797 patients with tumors classified with AJCC stage 0, I or II and and 725 patients had complete information for age, gender and Breslow thickness (Table 1). Spanish melanoma patients were recruited at the Department of Dermatology, Instituto Valenciano de Oncologia, a referral skin cancer centre for the provinces of Valencia, Alicante, and Castellón, with a catchment population of ∼5 million people. Blood samples from melanoma patients were collected between 2000 and 2007 and diagnoses were confirmed by histopathology. The ethical approval for the study was granted by Ethics Commission of the Faculty of Clinical Medicine of University of Heidelberg and written informed consent was obtained from all study participants. All the patients were of European ethnicity.

**Table 1:** Characteristics of the melanoma patients from Germany and Spain.

	GERMANY			SPAIN		
	All patients with skin melanoma	Patients with AJCC stage 0, I or II at first diagnosis (FD)	Patients with AJCC stage 0, I or II at FD and complete information for Age, Gender and Breslow thickness	All patients with skin melanoma	Patients with AJCC stage 0, I or II at first diagnosis (FD)	Patients with AJCC stage 0, I or II at FD and complete information for Age, Gender and Breslow thickness
***Number of patients***	752	625	541	837	710	638
***Gender***						
**Male**	412 (54.8%)	341 (54.6%)	301 (55.6%)	379 (45.3%)	309 (43.5%)	281 (44.0%)
**Female**	340 (45.2%)	284 (45.4%)	240 (44.4%)	458 (54.7%)	401 (56.5%)	357 (56.0%)
***Age at FD***						
**Median**	55	55	55	53	52	52
**Mean**	54.0	54.0	54.4	51.5	51.0	50.8
**Standard deviation**	16.0	15.9	15.7	16.0	16.0	16.0
***Breslow thickness (mm)***						
**Median**	−	−	1.2	–	–	1.0
**Mean (95%CI)**	−	−	1.8 (1.6–1.9)	–	–	1.6 (1.5–1.8)
**Standard deviation**	−	−	1.8	–	–	1.9
**Range (minimum, maximum)**	−	−	14.0 (0.0, 14.0)	–	–	17.9 (0.1, 18.0)
***AJCC stages at FD***						
**0**	10 (1.3%)	10 (1.6%)	6 (1.1%)	70 (8.4%)	70 (8.8%)	4 (0.6%)
**I**	400 (53.2%)	400 (64.0%)	338 (62.5%)	451 (53.9%)	451 (56.7%)	446 (69.9%)
**II**	215 (28.6%)	215 (34.4%)	197 (36.4%)	189 (22.6%)	189 (23.7%)	188 (29.5%)
**III**	111 (14.8%)	–	–	114 (13.6%)	–	–
**IV**	11 (1.5%)	–	–	4 (0.5%)	–	–
**unknown**	5 (0.7%)	-	–	10 (1.2%)	–	–
***Total number of metastasis***	379 (50.4%)	257 (41.1%)	218 (40.3%)	146 (17.4%)	92 (12.9%)	87 (13.6%)
***Total number of deaths***	238 (31.6%)	174 (27.8%)	146 (27.0%)	77 (9.2%)	45 (6.3%)	45 (7.1%)

### Selection of Polymorphisms in Interferon Gene Cluster on Chromosome 9p22

**Figure 1:**
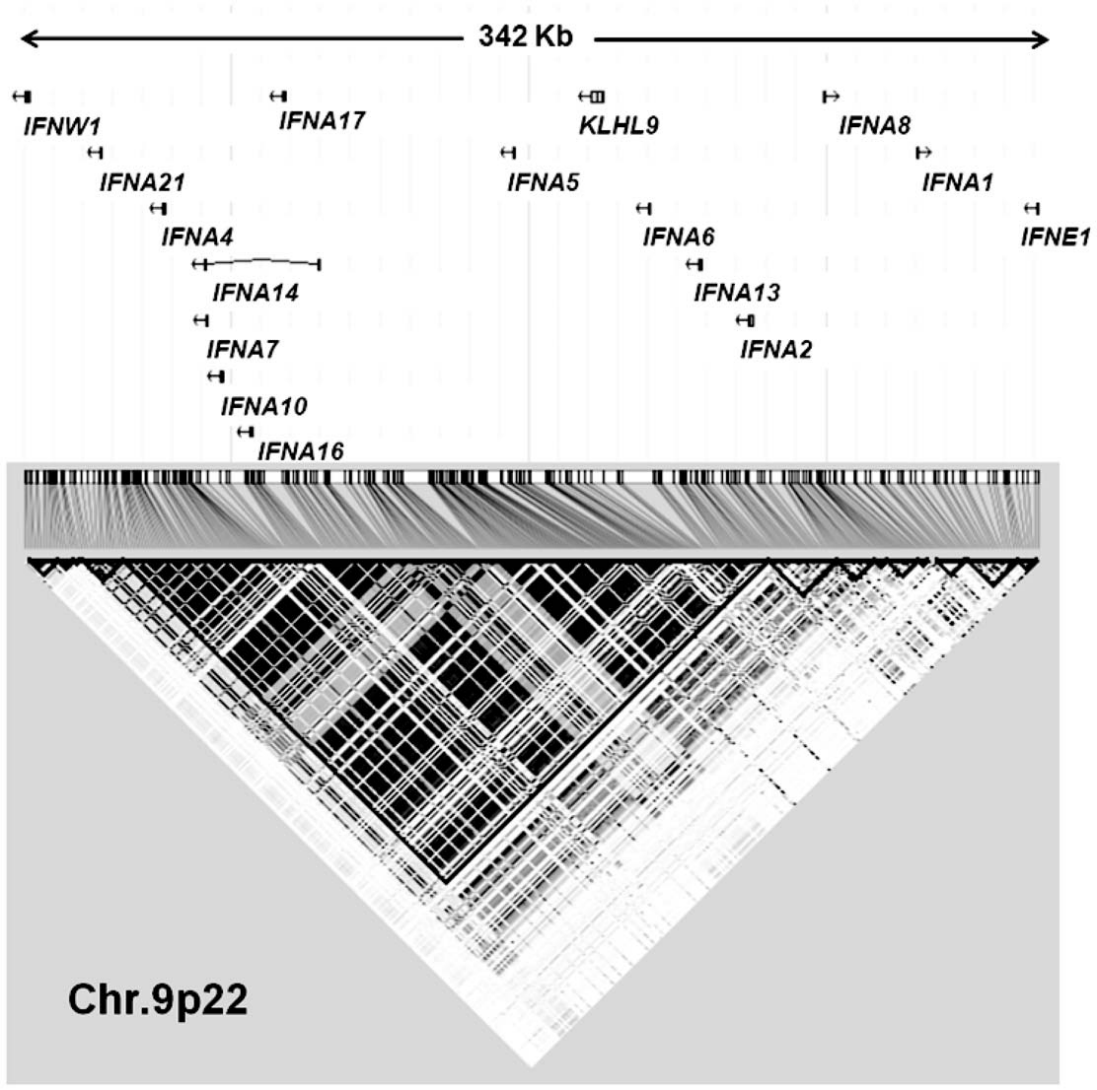
Type I interferons cluster region studied on chromosome 9p22.

We selected 44 SNPs using tagging approach, which encompassed 15 genes and represented 299 SNPs tagged with a rˆ2≥0.8 (Table S1). We aimed to evaluate an entire set of variants within a 342 kb region that contained a cluster of type I interferon genes on chromosome 9p22. *IFNW1*, located 24 kb farther from *IFNA21*, and *IFNE*, located at a distance of 40 kb from *IFNA1*, defined the limits of the locus. We included SNPs within the *KLH9* gene because of its location within the type I interferon cluster (Figure 1). The inclusion criteria also included a minor allele frequency (MAF) of 5% or more in Caucasian population based on HapMap data (release #28).

### Genotyping

Genotyping was carried out using an allelic discrimination method (Kaspar Assay from KBiosciences). PCR was carried out in 384-well format in a total volume of 4µl using 5 ng of DNA template, 0.11µl of assay mix (100 nM of two allele specific forward primers and common reverse primer, final concentration), 4µl reaction mix (Kbiosciences) and MgCl_2_. The reactions were performed using standard optimized conditions. Undetermined samples were genotyped again separately in a 96-well plate format. For quality control, ∼5% of samples were randomly selected and included as replicates.

### Statistical Analysis

Survival analyses were performed for the polymorphisms within the interferon genes in order to investigate the influence of the genotypes on disease free survival (DFS), time from metastasis to death (MD) and overall survival (OS). DFS was defined as the time (in years) from diagnosis of primary melanoma until the first metastasis (either regional or distant). MD survival was defined as the time from first metastasis to death or last patient contact. For MD analysis we only considered those patients who developed metastasis during the follow up time. The OS was the time from date of first diagnosis until death or last patient contact. The associations of the genotypes with DFS, MD and OS were estimated for 10 years using Kaplan-Meier methods and log-rank test was used to compare difference between the survival curves. Haplotype frequencies were inferred using the expectation-maximization algorithm (PROC HAPLOTYPE, SAS/Genetics Software). For the genotypes and inferred haplotypes associations were estimated as hazard ratio (HR) based on Cox regression, adjusted for gender, age and Breslow thickness (PROC PHREG, SAS 9.2). The analysis was carried out on data from the German and Spanish patients without metastasis at first diagnosis (AJCC stages 0, I or II) with adjustment for age, gender and Breslow thickness (Tables S2 and S3). Ulceration status was not included in the multivariate analysis due to unavailability of complete data.

### Determination of the Effect of Genotypes on Melanoma Therapy

Genotype data were also analyzed after stratification of both German and Spanish melanoma patients on the basis of administered therapy; survival analyses were performed on different subgroups of patients, separately. Start time and duration of therapy were considered to explore the possible interaction between therapy and genotypes. The treatment was taken into account in statistics analysis as a time-dependent variable. The variability in treatment in terms of dosage and frequency could not be evaluated due to lack of information.

**Figure 2:**
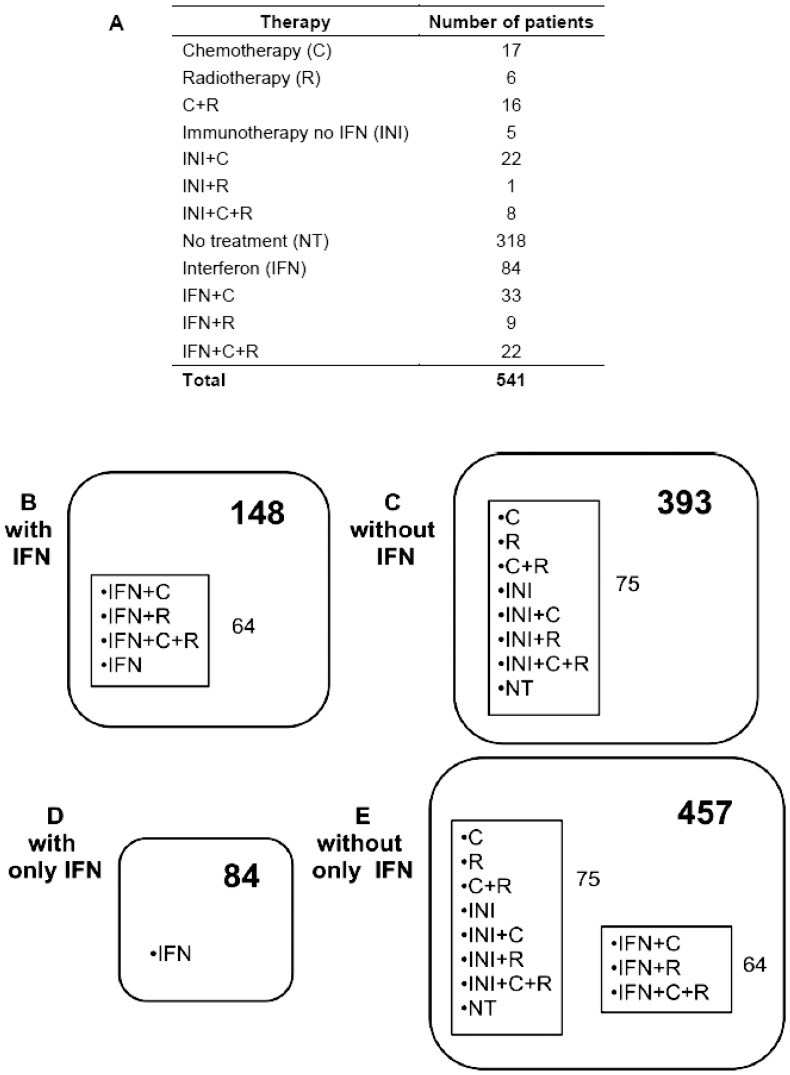
A, Number of melanoma patients from Germany and therapy types. B, ”with IFN”, patients that received IFN alone or combined with other treatments. C, ”without IFN”, patients that either did not receive any therapy or that received different kinds of therapies but never IFN. D, ”with only IFN”, patients that received only IFN as therapy. E, ”without only IFN”, patients that either did not receive any therapy or that received different kinds of therapies combined or not with IFN. IFN, interferon; C, chemotherapy; R, radiotherapy; INI, immunotherapy no IFN; NT, no treatment.

German melanoma patients included those who did not receive any therapy or those who received different kinds of therapies including chemotherapy, radiotherapy and/or immunotherapy. Within the immune-treated group there were those patients that received interferon as adjuvant therapy, and those patients that received different types of immune-treatment in stage IV as part of an chemoimmunotherapy regimen (Figure 2 A). We analyzed data after stratification into groups, those who received interferon, alone or in combination with some other kind of treatment (chemotherapy and/or radiotherapy), and the rest of the patients that never received interferon. We called these two groups “with interferon” and “without interferon”, respectively (Figure 2 B and C). We also compared a subgroup of 84 patients that received only interferon and no other kind of treatment with the total set of the patients without therapy and those that went under different therapies without only IFN. We called these groups “with only interferon” and “without only interferon”, respectively (Figure 2 D and E).

Spanish melanoma patient group was comprised of cases those received interferon as adjuvant treatment and those who did not receive any kind of treatment. These patients were analyzed under similar criteria of “with only interferon” and “without only interferon”, with the difference being that, unlike the patients from Germany, in the “without only interferon” group, the Spanish melanoma patients received no treatment at all.

### Prediction of Functional Effects of Human SNPs Located at the 5′ and 3′UTR

Prediction models were used for the role in putative miRNA-mRNA interactions of those polymorphisms that were associated with different melanoma survival parameters and were located within and near 3′UTRs of the genes. Different algorithms were used to predict the ability of the variant to affect miRNA binding sites, which included PITA (http://genie.weizmann.ac.il/pubs/mir07/mir07_prediction.html) [24], PolymiRTS (Polymorphism in microRNA Target Site) Database 2.0 (http://compbio.uthsc.edu/miRSNP/search.php) [25]–[27], and miRanda (http://www.microrna.org/microrna/getGeneForm.do) [27], [28]. Similarly, in order to identify putative transcription factor binding sites at sequence motifs containing melanoma survival associated SNPs, located near 5′UTRs of the genes, we used the TESS (Transcription Element Search System) web-based software tool (http://www.cbil.upenn.edu/cgi-bin/tess) [29].

## Results

### Results of Genotype Analysis of Polymorphisms in Interferon Genes

We genotyped 625 melanoma patients recruited in an oncology center in Germany for 44 polymorphisms located on chromosome 9p22 that were informative for 299 polymorphisms and spanned 15 type I interferon genes. Data analysis showed statistical significant association between variants alleles of three polymorphisms, rs10964859 (3′UTR of *IFNW1*), rs10964862 and rs597408, in more than one survival parameter (Table 2). The three polymorphisms were additionally genotyped in an independent set of 797 melanoma patients recruited in Valencia, Spain, and the data confirmed the association between the rs10964859 and rs10964862 polymorphisms and different survival parameters similar to that in German patients. The association observed for the rs597408 polymorphism in German melanoma patients did not replicate in Spanish patients.

**Table 2:** Estimated 10 years survival analysis performed on the patients from Germany with first metastasis at diagnosis adjusted for age, gender and Breslow thickness.

		Overall survival						Disease free progression						Metastasis to death					
SNP	genotype	n	D	%	HR	CI	P	n	M	%	HR	CI	P	n	D	%	HR	CI	P
**rs1424860**	TT	385	90	23.40	1.00	(referent)	-	385	141	36.60	1.00	(referent)	-	155	100	64.50	1.00	(referent)	-
	TC	138	26	18.80	0.63	(0.40 - 1.00)	**0.05**	138	52	37.70	0.89	(0.64 - 1.24)	0.49	57	30	52.60	0.68	(0.45 - 1.03)	0.07
	CC	8	2	25.00	0.94	(0.23 - 3.85)	0.94	8	3	37.50	1.04	(0.33 - 3.26)	0.95	4	3	75.00	1.36	(0.43 - 4.32)	0.60
	TC +CC	146	28	19.20	0.65	(0.42 - 1.01)	0.06	146	55	37.70	0.90	(0.65 - 1.24)	0.51	61	33	54.10	0.71	(0.48 - 1.07)	0.10
**rs10964859**	CC	232	43	18.50	1.00	(referent)	-	232	72	31.00	1.00	(referent)	-	84	51	60.70	1.00	(referent)	-
	CG	247	57	23.10	1.16	(0.77 - 1.73)	0.48	247	97	39.30	1.29	(0.94 - 1.76)	0.11	104	62	59.60	1.00	(0.68 - 1.46)	1.00
	GG	57	17	29.80	1.80	(1.02 - 3.16)	**0.04**	57	26	45.60	1.51	(0.96 - 2.37)	0.07	27	20	74.10	2.01	(1.17 - 3.44)	**0.01**
	CG +GG	304	74	24.30	1.27	(0.87 - 1.86)	0.22	304	123	40.50	1.33	(0.99 - 1.79)	0.06	131	82	62.60	1.14	(0.80 - 1.63)	0.46
**rs10511694**	CC	267	65	24.30	1.00	(referent)	-	267	105	39.30	1.00	(referent)	-	114	76	66.70	1.00	(referent)	-
	TC	219	43	19.60	0.80	(0.54 - 1.19)	0.28	219	76	34.70	0.95	(0.70 - 1.29)	0.75	85	47	55.30	0.64	(0.44 - 0.92)	**0.02**
	TT	52	10	19.20	0.74	(0.38 - 1.46)	0.39	52	15	28.80	0.67	(0.39 - 1.16)	0.15	17	10	58.80	0.63	(0.32 - 1.23)	0.18
	TC +TT	271	53	19.60	0.79	(0.55 - 1.15)	0.22	271	91	33.60	0.89	(0.67 - 1.19)	0.43	102	57	55.90	0.64	(0.45 - 0.90)	**0.01**
**rs2081381**	CC	197	44	22.30	1.00	(referent)	-	197	75	38.10	1.00	(referent)	-	83	50	60.20	1.00	(referent)	-
	CG	264	59	22.30	1.07	(0.72 - 1.59)	0.74	264	101	38.30	1.02	(0.75 - 1.37)	0.92	107	65	60.70	1.21	(0.83 - 1.75)	0.33
	GG	65	13	20.00	1.02	(0.55 - 1.90)	0.95	65	18	27.70	0.74	(0.44 - 1.25)	0.26	24	17	70.80	1.21	(0.69 - 2.12)	0.51
	CG +GG	329	72	21.90	1.06	(0.72 - 1.55)	0.77	329	119	36.20	0.96	(0.72 - 1.29)	0.79	131	82	62.60	1.21	(0.85 - 1.72)	0.30
**rs10081742**	AA	396	81	20.50	1.00	(referent)	-	396	136	34.30	1.00	(referent)	-	150	88	58.70	1.00	(referent)	-
	AG	124	35	28.20	1.54	(1.03 - 2.31)	**0.04**	124	55	44.40	1.47	(1.07 - 2.03)	**0.02**	59	42	71.20	1.43	(0.97 - 2.10)	0.07
	GG	7	-	-	-	-	-	7	3	42.90	0.74	(0.23 - 2.38)	0.62	4	-	-	-	-	-
	AG +GG	131	35	26.70	1.31	(0.87 - 1.96)	0.19	131	58	44.30	1.40	(1.03 - 1.91)	**0.03**	63	42	66.70	1.32	(0.90 - 1.94)	0.16
**rs10964862**	CC	231	43	18.60	1.00	(referent)	-	231	69	29.90	1.00	(referent)	-	80	47	58.80	1.00	(referent)	-
	CA	253	60	23.70	1.12	(0.75 - 1.69)	0.58	253	104	41.10	1.38	(1.01 - 1.88)	**0.04**	112	69	61.60	1.04	(0.71 - 1.52)	0.85
	AA	49	14	28.60	1.67	(0.91 - 3.07)	0.10	49	23	46.90	1.63	(1.01 - 2.62)	**0.04**	24	17	70.80	1.84	(1.03 - 3.27)	**0.04**
	CA +AA	302	74	24.50	1.20	(0.82 - 1.78)	0.35	302	127	42.10	1.42	(1.05 - 1.91)	**0.02**	136	86	63.20	1.14	(0.79 - 1.64)	0.50
**rs10964863**	CC	217	49	22.60	1.00	(referent)	-	217	83	38.20	1.00	(referent)	-	93	58	62.40	1.00	(referent)	-
	CT	258	55	21.30	1.02	(0.70 - 1.51)	0.90	258	100	38.80	1.01	(0.76 - 1.36)	0.93	106	60	56.60	0.98	(0.68 - 1.41)	0.90
	TT	49	11	22.40	1.15	(0.60 - 2.22)	0.68	49	10	20.40	0.53	(0.27 - 1.02)	0.06	14	12	85.70	1.58	(0.84 - 2.97)	0.15
	CT +TT	307	66	21.50	1.04	(0.72 - 1.51)	0.82	307	110	35.80	0.93	(0.70 - 1.24)	0.64	120	72	60.00	1.04	(0.74 - 1.48)	0.81
**rs10811482**	AA	319	71	22.30	1.00	(referent)	-	319	109	34.20	1.00	(referent)	-	123	77	62.60	1.00	(referent)	-
	AG	196	39	19.90	0.83	(0.56 - 1.24)	0.37	196	75	38.30	1.07	(0.79 - 1.44)	0.68	81	48	59.30	0.98	(0.68 - 1.41)	0.90
	GG	18	7	38.90	2.07	(0.94 - 4.56)	0.07	18	10	55.60	1.65	(0.86 - 3.19)	0.14	10	7	70.00	1.79	(0.81 - 3.92)	0.15
	AG +GG	214	46	21.50	0.92	(0.63 - 1.34)	0.66	214	85	39.70	1.11	(0.83 - 1.48)	0.47	91	55	60.40	1.04	(0.73 - 1.48)	0.83
**rs7038852**	AA	180	41	22.80	1.00	(referent)	-	180	64	35.60	1.00	(referent)	-	70	44	62.90	1.00	(referent)	-
	AG	266	54	20.30	1.01	(0.67 - 1.52)	0.96	266	95	35.70	1.13	(0.82 - 1.55)	0.47	107	64	59.80	0.89	(0.60 - 1.30)	0.54
	GG	77	20	26.00	1.45	(0.84 - 2.49)	0.18	77	34	44.20	1.74	(1.14 - 2.67)	**0.01**	35	22	62.90	1.00	(0.60 - 1.68)	1.00
	AG +GG	343	74	21.60	1.10	(0.75 - 1.61)	0.64	343	129	37.60	1.23	(0.91 - 1.67)	0.17	142	86	60.60	0.91	(0.63 - 1.31)	0.62
**rs4568676**	CC	189	43	22.80	1.00	(referent)	-	189	73	38.60	1.00	(referent)	-	79	48	60.80	1.00	(referent)	-
	CG	277	59	21.30	1.00	(0.67 - 1.48)	0.99	277	95	34.30	0.86	(0.64 - 1.17)	0.35	105	65	61.90	1.09	(0.75 - 1.58)	0.67
	GG	66	15	22.70	0.91	(0.50 - 1.65)	0.76	66	26	39.40	0.90	(0.57 - 1.41)	0.64	30	19	63.30	1.27	(0.74 - 2.17)	0.39
	CG +GG	343	74	21.60	0.98	(0.67 - 1.43)	0.91	343	121	35.30	0.87	(0.65 - 1.16)	0.35	135	84	62.20	1.12	(0.78 - 1.61)	0.53
**rs2383183**	TT	437	93	21.30	1.00	(referent)	-	437	160	36.60	1.00	(referent)	-	178	108	60.70	1.00	(referent)	-
	TC	90	23	25.60	1.09	(0.68 - 1.73)	0.73	90	33	36.70	0.93	(0.63 - 1.36)	0.70	35	23	65.70	1.13	(0.71 - 1.78)	0.61
	CC	9	2	22.20	0.77	(0.19 - 3.14)	0.72	9	3	33.30	0.88	(0.28 - 2.77)	0.83	3	2	66.70	0.80	(0.19 - 3.27)	0.75
	TC +CC	99	25	25.30	1.05	(0.67 - 1.65)	0.83	99	36	36.40	0.92	(0.64 - 1.33)	0.67	38	25	65.80	1.09	(0.70 - 1.69)	0.70
**rs10119910**	AA	329	72	21.90	1.00	(referent)	-	329	122	37.10	1.00	(referent)	-	134	82	61.20	1.00	(referent)	-
	AT	180	40	22.20	0.95	(0.64 - 1.39)	0.78	180	65	36.10	0.95	(0.71 - 1.29)	0.76	73	44	60.30	0.84	(0.58 - 1.22)	0.37
	TT	19	4	21.10	0.88	(0.32 - 2.40)	0.80	19	5	26.30	0.63	(0.26 - 1.55)	0.32	5	3	60.00	0.96	(0.30 - 3.08)	0.95
	AT +TT	199	44	22.10	0.94	(0.64 - 1.37)	0.74	199	70	35.20	0.92	(0.69 - 1.24)	0.59	78	47	60.30	0.85	(0.59 - 1.22)	0.38
**rs10964912**	AA	292	63	21.60	1.00	(referent)	-	292	103	35.30	1.00	(referent)	-	111	68	61.30	1.00	(referent)	-
	AC	213	50	23.50	1.16	(0.80 - 1.69)	0.43	213	82	38.50	1.21	(0.90 - 1.62)	0.20	92	56	60.90	0.97	(0.68 - 1.39)	0.88
	CC	30	5	16.70	0.59	(0.23 - 1.50)	0.27	30	10	33.30	0.64	(0.33 - 1.23)	0.18	11	8	72.70	1.78	(0.84 - 3.77)	0.14
	AC +CC	243	55	22.60	1.07	(0.75 - 1.54)	0.71	243	92	37.90	1.10	(0.83 - 1.46)	0.51	103	64	62.10	1.03	(0.73 - 1.46)	0.86
**rs13340713**	CC	495	109	22.00	1.00	(referent)	-	495	181	36.60	1.00	(referent)	-	198	122	61.60	1.00	(referent)	-
	CG	39	8	20.50	0.81	(0.39 - 1.68)	0.58	39	14	35.90	0.88	(0.51 - 1.52)	0.64	17	10	58.80	0.89	(0.46 - 1.70)	0.72
	CG +GG	39	8	20.50	0.81	(0.39 - 1.68)	0.58	39	14	35.90	0.88	(0.51 - 1.52)	0.64	17	10	58.80	0.89	(0.46 - 1.70)	0.72
**rs1330320**	TT	197	43	21.80	1.00	(referent)	-	197	71	36.00	1.00	(referent)	-	76	47	61.80	1.00	(referent)	-
	TC	249	56	22.50	0.87	(0.58 - 1.31)	0.51	249	96	38.60	1.01	(0.74 - 1.38)	0.94	107	66	61.70	0.92	(0.63 - 1.35)	0.68
	CC	85	18	21.20	0.89	(0.51 - 1.54)	0.67	85	28	32.90	0.88	(0.57 - 1.37)	0.57	32	19	59.40	0.88	(0.51 - 1.51)	0.64
	TC +CC	334	74	22.20	0.88	(0.60 - 1.28)	0.49	334	124	37.10	0.98	(0.73 - 1.31)	0.88	139	85	61.20	0.91	(0.63 - 1.31)	0.62
**rs10757212**	GG	333	72	21.60	1.00	(referent)	-	333	122	36.60	1.00	(referent)	-	134	83	61.90	1.00	(referent)	-
	GA	184	42	22.80	1.01	(0.69 - 1.48)	0.97	184	70	38.00	1.04	(0.77 - 1.40)	0.80	78	48	61.50	0.88	(0.61 - 1.27)	0.51
	AA	18	3	16.70	0.70	(0.22 - 2.22)	0.54	18	4	22.20	0.55	(0.20 - 1.48)	0.24	4	2	50.00	0.73	(0.18 - 3.00)	0.66
	GA +AA	202	45	22.30	0.98	(0.67 - 1.42)	0.91	202	74	36.60	0.99	(0.74 - 1.32)	0.95	82	50	61.00	0.87	(0.61 - 1.25)	0.46
**rs7031048**	AA	362	79	21.80	1.00	(referent)	-	362	135	37.30	1.00	(referent)	-	149	92	61.70	1.00	(referent)	-
	AG	163	36	22.10	0.97	(0.65 - 1.43)	0.86	163	57	35.00	0.89	(0.66 - 1.22)	0.48	63	39	61.90	0.91	(0.62 - 1.34)	0.64
	GG	13	3	23.10	1.06	(0.33 - 3.38)	0.92	13	4	30.80	0.87	(0.32 - 2.38)	0.79	4	2	50.00	0.74	(0.18 - 3.04)	0.68
	AG +GG	176	39	22.20	0.97	(0.66 - 1.43)	0.89	176	61	34.70	0.89	(0.66 - 1.21)	0.47	67	41	61.20	0.90	(0.62 - 1.31)	0.59
**rs3758236**	TT	363	79	21.80	1.00	(referent)	-	363	135	37.20	1.00	(referent)	-	150	93	62.00	1.00	(referent)	-
	TA	158	36	22.80	1.03	(0.69 - 1.53)	0.89	158	55	34.80	0.93	(0.68 - 1.27)	0.65	60	38	63.30	0.93	(0.63 - 1.37)	0.71
	AA	11	2	18.20	0.75	(0.18 - 3.05)	0.68	11	4	36.40	0.99	(0.36 - 2.69)	0.99	4	2	50.00	0.75	(0.18 - 3.07)	0.69
	TA +AA	169	38	22.50	1.01	(0.68 - 1.49)	0.97	169	59	34.90	0.93	(0.69 - 1.27)	0.66	64	40	62.50	0.91	(0.63 - 1.34)	0.65
**rs913931**	AA	405	89	22.00	1.00	(referent)	-	405	154	38.00	1.00	(referent)	-	171	105	61.40	1.00	(referent)	-
	AG	112	26	23.20	1.06	(0.68 - 1.64)	0.80	112	35	31.30	0.73	(0.51 - 1.06)	0.10	38	25	65.80	1.23	(0.79 - 1.90)	0.36
	GG	8	2	25.00	1.62	(0.40 - 6.62)	0.50	8	4	50.00	2.11	(0.78 - 5.71)	0.14	4	2	50.00	0.88	(0.21 - 3.62)	0.86
	AG +GG	120	28	23.30	1.09	(0.71 - 1.66)	0.71	120	39	32.50	0.79	(0.55 - 1.12)	0.19	42	27	64.30	1.19	(0.78 - 1.83)	0.42
**rs597408**	AA	466	101	21.70	1.00	(referent)	-	466	169	36.30	1.00	(referent)	-	185	115	62.20	1.00	(referent)	-
	AG	50	9	18.00	0.89	(0.45 - 1.77)	0.75	50	17	34.00	0.95	(0.57 - 1.56)	0.83	21	10	47.60	0.87	(0.46 - 1.67)	0.68
	GG	14	7	50.00	2.75	(1.26 - 6.00)	**0.01**	14	8	57.10	2.16	(1.05 - 4.43)	**0.04**	8	7	87.50	1.79	(0.82 - 3.93)	0.15
	AG +GG	64	16	25.00	1.27	(0.75 - 2.15)	0.38	64	25	39.10	1.16	(0.76 - 1.76)	0.50	29	17	58.60	1.10	(0.66 - 1.84)	0.71
**rs10448208**	GG	426	94	22.10	1.00	(referent)	-	426	160	37.60	1.00	(referent)	-	176	106	60.20	1.00	(referent)	-
	GA	92	22	23.90	0.95	(0.59 - 1.51)	0.81	92	32	34.80	0.78	(0.53 - 1.14)	0.20	1	-	-	-	-	-
	AA	4	-	-	-	-	-	4	1	25.00	1.04	(0.15 - 7.48)	0.97	36	25	69.40	1.09	(0.70 - 1.70)	0.70
	GA +AA	96	22	22.90	0.93	(0.58 - 1.48)	0.75	96	33	34.40	0.78	(0.54 - 1.14)	0.20	37	25	66.70	1.09	(0.70 - 1.70)	0.70
**rs647167**	TT	382	85	22.30	1.00	(referent)	-	382	146	38.20	1.00	(referent)	-	162	101	62.30	1.00	(referent)	-
	TG	146	32	21.90	0.90	(0.60 - 1.36)	0.63	146	48	32.90	0.85	(0.61 - 1.18)	0.33	52	31	59.60	0.74	(0.49 - 1.12)	0.16
	GG	7	-	-	-	-	-	7	1	14.30	0.46	(0.06 - 3.32)	0.45	1	-	-	-	-	-
	TG +GG	153	32	20.90	0.87	(0.58 - 1.31)	0.51	153	49	32.00	0.83	(0.60 - 1.16)	0.28	53	31	58.50	0.72	(0.48 - 1.09)	0.12
**rs615544**	CC	413	93	22.50	1.00	(referent)	-	413	157	38.00	1.00	(referent)	-	175	109	62.30	1.00	(referent)	-
	CA	109	25	22.90	0.90	(0.58 - 1.42)	0.66	109	37	33.90	0.88	(0.61 - 1.26)	0.48	39	24	61.50	0.79	(0.50 - 1.24)	0.31
	AA	5	-	-	-	-	1.00	5	1	20.00	0.74	(0.10 - 5.27)	0.76	1	-	-	-	-	-
	CA +AA	114	25	21.90	0.87	(0.56 - 1.37)	0.55	114	38	33.30	0.87	(0.61 - 1.25)	0.46	40	24	60.00	0.76	(0.49 - 1.20)	0.24
**rs10120977**	AA	329	72	21.90	1.00	(referent)	-	329	119	36.20	1.00	(referent)	-	127	77	60.60	1.00	(referent)	-
	AG	187	43	23.00	1.12	(0.77 - 1.65)	0.55	187	71	38.00	1.06	(0.79 - 1.43)	0.68	82	53	64.60	1.20	(0.84 - 1.72)	0.31
	GG	21	3	14.30	0.83	(0.26 - 2.64)	0.75	21	5	23.80	0.72	(0.29 - 1.78)	0.48	6	2	33.30	0.78	(0.19 - 3.19)	0.73
	AG +GG	208	46	22.10	1.10	(0.76 - 1.60)	0.62	208	76	36.50	1.03	(0.77 - 1.38)	0.83	88	55	62.50	1.18	(0.83 - 1.67)	0.36
**rs632941**	GG	272	59	21.70	1.00	(referent)	-	272	102	37.50	1.00	(referent)	-	114	69	60.50	1.00	(referent)	-
	GA	238	56	23.50	0.96	(0.67 - 1.39)	0.84	238	90	37.80	0.93	(0.70 - 1.24)	0.63	98	63	64.30	0.90	(0.63 - 1.27)	0.55
	AA	28	3	10.70	0.48	(0.15 - 1.54)	0.22	28	4	14.30	0.35	(0.13 - 0.94)	**0.04**	4	1	25.00	0.48	(0.07 - 3.49)	0.47
	GA +AA	266	59	22.20	0.92	(0.64 - 1.32)	0.64	266	94	35.30	0.87	(0.66 - 1.15)	0.34	102	64	62.70	0.88	(0.62 - 1.25)	0.49
**rs1224391**	CC	203	45	22.20	1.00	(referent)	-	203	68	33.50	1.00	(referent)	-	74	49	66.20	1.00	(referent)	-
	TC	256	52	20.30	0.80	(0.53 - 1.21)	0.29	256	98	38.30	1.17	(0.86 - 1.60)	0.32	110	62	56.40	0.77	(0.53 - 1.13)	0.18
	TT	70	20	28.60	1.27	(0.75 - 2.16)	0.37	70	29	41.40	1.47	(0.95 - 2.27)	0.09	31	21	67.70	1.03	(0.62 - 1.73)	0.90
	TC +TT	326	72	22.10	0.90	(0.62 - 1.32)	0.59	326	127	39.00	1.23	(0.92 - 1.66)	0.17	141	83	58.90	0.83	(0.58 - 1.19)	0.30
**rs4978113**	TT	339	72	21.20	1.00	(referent)	-	339	129	38.10	1.00	(referent)	-	143	86	60.10	1.00	(referent)	-
	TC	179	42	23.50	0.98	(0.66 - 1.44)	0.90	179	62	34.60	0.83	(0.61 - 1.13)	0.24	68	44	64.70	0.98	(0.68 - 1.42)	0.93
	CC	11	1	9.10	0.37	(0.05 - 2.67)	0.32	11	1	9.10	0.22	(0.03 - 1.60)	0.14	1	-	-	-	-	-
	TC +CC	190	43	22.60	0.94	(0.64 - 1.38)	0.75	190	63	33.20	0.80	(0.59 - 1.08)	0.14	69	44	63.80	0.96	(0.67 - 1.39)	0.83
**rs1330322**	AA	177	40	22.60	1.00	(referent)	-	177	70	39.50	1.00	(referent)	-	75	44	58.70	1.00	(referent)	-
	AG	259	57	22.00	0.88	(0.58 - 1.33)	0.53	259	96	37.10	0.80	(0.58 - 1.10)	0.16	107	69	64.50	1.17	(0.80 - 1.72)	0.42
	GG	102	21	20.60	0.93	(0.55 - 1.58)	0.79	102	30	29.40	0.71	(0.46 - 1.08)	0.11	34	20	58.80	1.02	(0.60 - 1.74)	0.93
	AG +GG	361	78	21.60	0.89	(0.61 - 1.31)	0.56	361	126	34.90	0.77	(0.57 - 1.04)	0.09	141	89	63.10	1.13	(0.79 - 1.63)	0.51
**rs7025006**	GG	156	40	25.60	1.00	(referent)	-	156	61	39.10	1.00	(referent)	-	64	40	62.50	1.00	(referent)	-
	GA	273	54	19.80	0.68	(0.45 - 1.03)	0.07	273	95	34.80	0.75	(0.54 - 1.04)	0.09	107	68	63.60	1.19	(0.80 - 1.76)	0.39
	AA	103	23	22.30	0.84	(0.50 - 1.40)	0.50	103	37	35.90	0.83	(0.55 - 1.25)	0.38	42	24	57.10	0.92	(0.55 - 1.55)	0.77
	GA +AA	376	77	20.50	0.72	(0.49 - 1.06)	0.10	376	132	35.10	0.77	(0.57 - 1.05)	0.10	149	92	61.70	1.11	(0.76 - 1.61)	0.60
**rs2104880**	CC	479	111	23.20	1.00	(referent)	-	479	179	37.40	1.00	(referent)	-	198	125	63.10	1.00	(referent)	-
	CT	50	7	14.00	0.55	(0.25 - 1.17)	0.12	50	16	32.00	0.79	(0.47 - 1.32)	0.37	17	8	47.10	0.69	(0.34 - 1.42)	0.32
	TT	1	-	-	-	-	-	1	1	100.00	6.16	(0.82 - 46.1)	0.08	1	-	-	-	-	-
	CT +TT	51	7	13.70	0.55	(0.25 - 1.17)	0.12	51	17	33.30	0.83	(0.50 - 1.37)	0.47	18	8	44.40	0.69	(0.34 - 1.42)	0.32
**rs1332179**	TT	428	99	23.10	1.00	(referent)	-	428	160	37.40	1.00	(referent)	-	177	113	63.80	1.00	(referent)	-
	TC	104	19	18.30	0.71	(0.43 - 1.16)	0.17	104	34	32.70	0.81	(0.56 - 1.17)	0.26	1	-	-	-	-	-
	CC	5	-	-	-	-	-	5	1	20.00	0.68	(0.10 - 4.90)	0.70	37	20	54.10	0.77	(0.48 - 1.23)	0.27
	TC +CC	109	19	17.40	0.69	(0.42 - 1.13)	0.14	109	35	32.10	0.80	(0.56 - 1.16)	0.25	38	20	52.60	0.77	(0.48 - 1.23)	0.27
**rs1591032**	AA	352	81	23.00	1.00	(referent)	-	352	130	36.90	1.00	(referent)	-	145	92	63.40	1.00	(referent)	-
	AG	167	32	19.20	0.78	(0.52 - 1.18)	0.24	167	59	35.30	0.90	(0.66 - 1.23)	0.52	64	36	56.30	0.75	(0.51 - 1.11)	0.15
	GG	11	3	27.30	1.34	(0.42 - 4.29)	0.63	11	4	36.40	1.25	(0.46 - 3.40)	0.66	4	3	75.00	1.00	(0.31 - 3.19)	-
	AG +GG	178	35	19.70	0.81	(0.54 - 1.21)	0.30	178	63	35.40	0.92	(0.68 - 1.24)	0.59	68	39	57.40	0.77	(0.53 - 1.12)	0.17
**rs7871767**	GG	357	78	21.80	1.00	(referent)	-	357	132	37.00	1.00	(referent)	-	147	89	60.50	1.00	(referent)	-
	GA	143	31	21.70	0.92	(0.60 - 1.39)	0.69	143	49	34.30	0.84	(0.60 - 1.17)	0.30	51	34	66.70	1.08	(0.73 - 1.61)	0.70
	AA	8	-	-	-	-	-	8	2	25.00	0.77	(0.19 - 3.11)	0.71	2	-	-	-	-	-
	GA +AA	151	31	20.50	0.88	(0.58 - 1.33)	0.55	151	51	33.80	0.84	(0.60 - 1.16)	0.28	53	34	64.20	1.05	(0.71 - 1.57)	0.80
**rs7043990**	TT	409	96	23.50	1.00	(referent)	-	409	152	37.20	1.00	(referent)	-	169	110	65.10	1.00	(referent)	-
	TC	118	22	18.60	0.83	(0.52 - 1.33)	0.44	118	42	35.60	1.00	(0.71 - 1.41)	0.99	45	23	51.10	0.63	(0.40 - 1.00)	**0.05**
	CC	3	-	-	-	-	-	3	1	33.30	1.10	(0.15 - 7.93)	0.92	1	-	-	-	-	-
	TC +CC	121	22	18.70	0.81	(0.51 - 1.29)	0.37	121	43	35.50	1.00	(0.71 - 1.41)	0.98	46	23	50.00	0.61	(0.39 - 0.96)	**0.03**
**rs12337364**	CC	446	105	23.50	1.00	(referent)	-	446	165	37.00	1.00	(referent)	-	182	118	64.80	1.00	(referent)	-
	CT	81	13	16.00	0.77	(0.43 - 1.38)	0.39	81	30	37.00	1.14	(0.77 - 1.69)	0.52	33	15	45.50	0.65	(0.38 - 1.11)	0.11
	TT	2	-	-	-	-	-	2	-	-	-	-	-	-	-	-	-	-	-
	CT +TT	83	13	15.70	0.76	(0.42 - 1.36)	0.35	83	30	36.10	1.11	(0.75 - 1.64)	0.61	33	15	45.50	0.65	(0.38 - 1.11)	0.11
**rs1332190**	TT	266	62	23.30	1.00	(referent)	-	266	95	35.70	1.00	(referent)	-	107	69	64.50	1.00	(referent)	-
	TC	227	50	22.00	0.86	(0.59 - 1.25)	0.42	227	89	39.20	1.02	(0.76 - 1.36)	0.92	97	59	60.80	0.84	(0.59 - 1.19)	0.33
	CC	45	6	13.30	0.50	(0.22 - 1.18)	0.11	45	12	26.70	0.69	(0.38 - 1.27)	0.23	12	5	41.70	0.56	(0.22 - 1.39)	0.21
	TC +CC	272	56	20.60	0.80	(0.56 - 1.15)	0.22	272	101	37.10	0.96	(0.73 - 1.27)	0.78	109	64	58.70	0.81	(0.57 - 1.13)	0.22
**rs7864960**	GG	131	36	27.50	1.00	(referent)	-	131	46	35.10	1.00	(referent)	-	51	32	62.70	1.00	(referent)	-
	GA	274	53	19.30	0.58	(0.38 - 0.91)	**0.02**	274	101	36.90	0.89	(0.62 - 1.27)	0.52	113	66	58.40	0.99	(0.65 - 1.52)	0.96
	AA	126	27	21.40	0.70	(0.42 - 1.15)	0.16	126	46	36.50	0.93	(0.61 - 1.39)	0.71	49	33	67.30	1.05	(0.64 - 1.72)	0.85
	GA +AA	400	80	20.00	0.62	(0.42 - 0.93)	**0.02**	400	147	36.80	0.90	(0.65 - 1.26)	0.55	162	99	61.10	1.01	(0.67 - 1.51)	0.97
**rs6475535**	CC	356	77	21.60	1.00	(referent)	-	356	132	37.10	1.00	(referent)	-	146	87	59.60	1.00	(referent)	-
	CG	168	40	23.80	1.00	(0.68 - 1.47)	1.00	168	62	36.90	0.92	(0.68 - 1.25)	0.62	68	45	66.20	1.05	(0.73 - 1.52)	0.79
	GG	7	1	14.30	0.50	(0.07 - 3.59)	0.49	7	2	28.60	0.58	(0.14 - 2.34)	0.44	2	1	50.00	0.80	(0.11 - 5.77)	0.82
	CG +GG	175	41	23.40	0.98	(0.67 - 1.43)	0.90	175	64	36.60	0.91	(0.67 - 1.23)	0.53	70	46	65.70	1.04	(0.73 - 1.50)	0.82
**rs10491569**	CC	266	65	24.40	1.00	(referent)	-	266	96	36.10	1.00	(referent)	-	103	67	65.00	1.00	(referent)	-
	CT	221	42	19.00	0.80	(0.54 - 1.18)	0.26	221	83	37.60	1.03	(0.77 - 1.39)	0.83	94	52	55.30	0.96	(0.66 - 1.40)	0.83
	TT	41	11	26.80	1.12	(0.59 - 2.12)	0.74	41	15	36.60	0.91	(0.53 - 1.57)	0.73	17	14	82.40	1.51	(0.84 - 2.70)	0.17
	CT +TT	262	53	20.20	0.85	(0.59 - 1.22)	0.38	262	98	37.40	1.01	(0.76 - 1.34)	0.94	111	66	59.50	1.05	(0.74 - 1.49)	0.80
**rs1888888**	GG	472	100	21.20	1.00	(referent)	-	472	171	36.20	1.00	(referent)	-	186	114	61.30	1.00	(referent)	-
	GA	62	17	27.40	1.39	(0.83 - 2.34)	0.21	62	22	35.50	1.02	(0.65 - 1.59)	0.95	27	18	66.70	1.39	(0.84 - 2.30)	0.19
	AA	1	1	100.00	20.79	(2.79 - 155 )	-	1	1	100.00	10.07	(1.39 - 73.2)	**0.02**	1	1	100.00	4.25	(0.58 - 31.4)	0.16
	GA +AA	63	17	27.00	1.47	(0.88 - 2.44)	0.14	63	23	36.50	1.06	(0.68 - 1.64)	0.80	28	18	64.30	1.44	(0.88 - 2.36)	0.14
**rs1412395**	AA	181	40	22.10	1.00	(referent)	-	181	68	37.60	1.00	(referent)	-	75	49	65.30	1.00	(referent)	-
	AG	266	53	19.90	1.00	(0.66 - 1.51)	1.00	266	92	34.60	1.05	(0.76 - 1.43)	0.78	102	58	56.90	0.94	(0.64 - 1.38)	0.76
	GG	80	22	27.50	1.12	(0.66 - 1.88)	0.68	80	29	36.30	0.97	(0.63 - 1.50)	0.89	32	23	71.90	1.09	(0.66 - 1.81)	0.73
	AG +GG	346	75	21.70	1.03	(0.70 - 1.52)	0.88	346	121	35.00	1.03	(0.76 - 1.38)	0.87	134	81	60.40	0.98	(0.68 - 1.41)	0.91
**rs2383192**	TT	132	28	21.20	1.00	(referent)	-	132	53	40.20	1.00	(referent)	-	58	36	62.10	1.00	(referent)	-
	TC	284	59	20.80	1.08	(0.69 - 1.70)	0.74	284	99	34.90	0.93	(0.66 - 1.31)	0.68	110	64	58.20	1.03	(0.68 - 1.55)	0.90
	CC	106	28	26.40	1.07	(0.63 - 1.82)	0.80	106	39	36.80	0.85	(0.56 - 1.29)	0.45	43	30	69.80	1.09	(0.66 - 1.78)	0.74
	TC +CC	390	87	22.30	1.08	(0.70 - 1.66)	0.73	390	138	35.40	0.91	(0.66 - 1.25)	0.55	153	94	61.40	1.05	(0.71 - 1.54)	0.82
**rs1125488**	AA	469	99	21.10	1.00	(referent)	-	469	171	36.50	1.00	(referent)	-	188	112	59.60	1.00	(referent)	-
	AC	64	18	28.10	1.16	(0.70 - 1.92)	0.57	64	25	39.10	1.03	(0.68 - 1.58)	0.88	27	20	74.10	1.29	(0.79 - 2.10)	0.30
	CC	2	-	-	-	-	-	2	-	-	-	-	-	-	-	-	-	-	-
	AC +CC	66	18	27.30	1.15	(0.69 - 1.91)	0.58	66	25	37.90	1.02	(0.67 - 1.56)	0.94	27	20	74.10	1.29	(0.79 - 2.10)	0.30
**rs10811561**	AA	152	37	24.30	1.00	(referent)	-	152	58	38.20	1.00	(referent)	-	64	43	67.20	1.00	(referent)	-
	AG	289	57	19.70	0.87	(0.57 - 1.32)	0.51	289	103	35.60	1.10	(0.79 - 1.52)	0.58	112	62	55.40	0.79	(0.53 - 1.17)	0.25
	GG	90	23	25.60	1.00	(0.59 - 1.69)	1.00	90	31	34.40	1.00	(0.64 - 1.56)	1.00	35	26	74.30	0.96	(0.58 - 1.56)	0.86
	AG +GG	379	80	21.10	0.90	(0.61 - 1.34)	0.61	379	134	35.40	1.07	(0.78 - 1.47)	0.66	147	88	59.90	0.83	(0.58 - 1.21)	0.34

n, number of cases; M, number of metastasis; D, number of deaths; HR, hazard ratio; CI, confidence interval.

**Figure 3:**
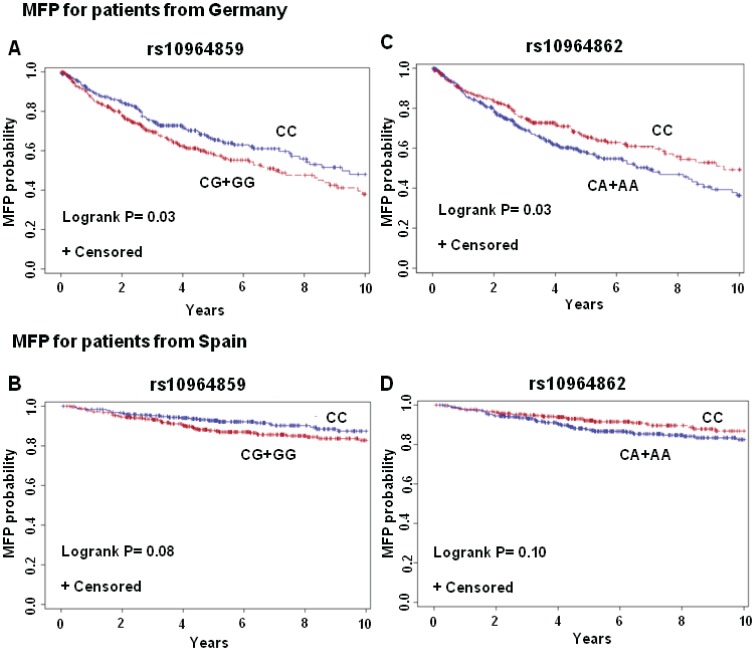
Survival plot for the major genotype and carriers of the minor genotype for rs10964859 and rs10964862 for DFS in the (A) German and (B) Spanish patients.

Data analysis showed that the carriers of the variant allele of the rs10964859 polymorphism were associated with a shorter time to the develop metastasis than the non-carriers (7.0 years versus 9.3 years; Kaplan-Meier survival log rank test P = 0.03; Figure 3 A). Multivariate Cox regression showed associated HR 1.33 for DFS in the carriers (95%CI 0.99–1.74; P = 0.06; Table S4). The patients, homozygote for minor G-allele, had a median survival of 2.0 years from metastasis to death compared to 4.2 years for patients that were homozygote for major C-allele (data not shown). The multivariate Cox regression showed that the carriers of the homozygous GG-genotype for rs10964859 were associated with HR 2.01 (95%CI 1.17–3.44; P = 0.01; Table S4) for MD and OS (HR 1.80; 95%CI 1.02–3.16; P = 0.04; Table S4). Analysis of data after stratification for the therapy showed that GG genotype of the rs10964859 polymorphism was associated with reduced DFS (HR = 1.61, 95% CI 0.99–2.62; P = 0.05) in the group of patients who never received only IFN (Table S5, Figure 2 E “without only IFN”).

The minor allele GG-genotype of rs10964859 was also associated with reduced time for MD in the group “without only IFN” (HR = 1.80, 95% CI 1.04–3.11; P = 0.04; Table S5, Figure 2 E). 78% of the patients with GG genotype had died within the group “with IFN” (HR = 3.08, a 95% CI 1.48–6.40; P = 0.003; Table S6, Figure 2 B) compared to 53% with the CC homozyous genotype. The effect of the minor allele GG genotype on the risk of death was higher with than without therapy based stratification.

The same group of patients with GG genotype showed association with OS (HR = 1.84, 95% CI 1.04–3.26; P = 0.04; Table S5). We observed that the detrimental effect of GG genotype on OS was mainly visible in the group that received either IFN therapy alone or in combination with other therapies (HR = 2.38, 95% CI 1.13–5.02; P = 0.02; Table S6, Figure 2 B “with IFN”). Within that group 57% of the patients with the GG homozygous genotype had died compared to 30% with the CC homozyous genotype.

The analysis of data from Spanish melanoma patients showed that carriers of the variant G-allele of the rs10964859 polymorphism were associated with an early development of metastasis compared to non-carriers (log rank test P = 0.08). The carriers of GG-genotype showed reduced time for DFS, reduced time for MD and decreased OS, but associations were not statistically significant. Detrimental effect of the variant allele was more pronounced in the carrier patients and who never received IFN therapy (HR 1.88; 95% 0.97–3.65; P = 0.06; Table S7, Figure 3 B).

The prediction analysis using PITA showed an intermediate ddG energy score for binding of hsa-mir-26a to the sequence motif containing the rs10964859 polymorphism. PolymiRTS algorithm predicted that CG nucleotide transition could influence the putative binding site of the hsa-miR-4503. It also indicated that the derived allele disrupts a conserved miRNA binding site.

The rs10964859 polymorphism was in linkage with 11 kb apart polymorphism rs10964862 with an r^2^ of 0.76. Melanoma patients with the variant A-allele of the rs10964862 polymorphism were associated with reduced time for DFS with 9.3 years versus 7.0 years (log rank test P = 0.03; Figure 3C) with multivariate HR 1.42 (95%CI 1.05–1.91; P = 0.02). The effect was statistically significant in heterozygote as well as homozygote carriers (Table 3). The patients homozygous for minor allele were also associated with reduced time for MD (HR 1.84, 95%CI 1.03–3.27, P = 0.04; Table 3). We also observed that the carriers of the minor allele of rs10964862 in the group of patients “without IFN” and “without only IFN” were associated with statistically significant increase in DFS rate (“without IFN” HR 1.52, 95%CI 1.02–2.28, P = 0.04; Table S8, Figure 2 C and “without only IFN” HR 1.48 95%CI 1.06–2.06, P = 0.02; Table S9, Figure 2 E). However the effect was not statistically significant in the groups of patients “with IFN” and “with only IFN”. We observed in the group “without only IFN”, patients homozygous for A-allele were at statistically increased risk of DFS (HR 1.78 95%CI 1.06–2.98; P = 0.03; Table S9). The minor allele AA genotype for rs10964862 was also statistically significantly associated with decreased MD survival only in the group “with IFN”, (HR of 2.62, 95% CI, 1.19–5.73; P = 0.02; Table S8, Figure 2 B). The validation of results in patients from Spain showed the association of the carriers of the variant allele for the rs10964862 polymorphism with a reduced DFS; though association after adjustment was not statistically significant (HR 1.32 95%CI 0.83–2.10; P = 0.24 Table 3)**.** The effect of variant genotypes on OS and MD was also not statistically significant. In the Spanish patients, for rs10964862 polymorphism, we observed that detrimental effect was statistically significant for the minor allele homozygous genotype for “without only IFN” patients (HR 2.52, 95%CI 1.07–5.90; P = 0.03; Table S10).

**Table 3:** Variation rs10964862 for the OS, DFS and MD analysis for the patients from Germany and Spain adjusted for the covariates age, gender and Breslow thickness.

rs10964862	genotype	cases	n	%	HR	CI	P
**OS GERMAN**	CC	231	43	18.6	1.00	(referent)	–
	CA	253	60	23.7	1.12	(0.75–1.69)	0.58
	AA	49	14	28.6	1.67	(0.91–3.07)	0.10
	CA +AA	302	74	24.5	1.20	(0.82–1.78)	0.35
**OS SPANISH**	CC	271	13	4.8	1.00	(referent)	–
	CA	295	20	6.8	1.39	(0.69–2.80)	0.36
	AA	67	7	10.4	2.13	(0.85–5.35)	0.11
	CA +AA	362	27	7.5	1.53	(0.79–2.97)	0.21
**DFS GERMAN**	CC	231	69	29.9	1.00	(referent)	–
	CA	253	104	41.1	1.38	(1.00–1.88)	**0.04**
	AA	49	23	46.9	1.63	(1.01–2.62)	**0.04**
	CA +AA	302	127	42.1	1.42	(1.05–1.91)	**0.02**
**DFS SPANISH**	CC	271	28	10.3	1.00	(referent)	–
	CA	295	38	12.9	1.24	(0.76–2.02)	0.39
	AA	67	11	16.4	1.69	(0.84–3.40)	0.14
	CA +AA	362	49	13.5	1.32	(0.83–2.10)	0.24
**MD GERMAN**	CC	80	47	58.8	1.00	(referent)	–
	CA	112	69	61.6	1.04	(0.71–1.52)	0.85
	AA	24	17	70.8	1.84	(1.03–3.27)	**0.04**
	CA +AA	136	86	63.2	1.14	(0.79–1.64)	0.50
**MD SPANISH**	CC	31	16	51.6	1.00	(referent)	–
	CA	44	20	45.5	1.02	(0.52–2.00)	0.97
	AA	12	7	58.3	1.36	(0.55–3.34)	0.51
	CA +AA	56	27	48.2	1.09	(0.58–2.06)	0.79

n, number of deaths for OS and MD analysis or number of metastases for DFS analysis.

OS, overall survival; DFS, disease free progression; MD, metastasis to death.

HR, Hazard Ratio; CI, Confidence Interval.

The patients with homozygous genotype for the variant G-allele for the rs597408 polymorphism were associated with DFS (HR 2.16, 95%CI 1.05–4.43, P = 0.04) and reduced OS (HR 2.75, 95%CI 1.26–6.00, P = 0.01; Table S11). After stratification, we also observed that detrimental effect was statistically significant for the minor allele genotype in the group “without only IFN” for the DFS (HR 2.09, 95%CI 1.01–4.29, P = 0.05) and OS analysis (HR 2.63, 95%CI 1.20–5.76, P = 0.02; Table S12). Though, results did not replicate in Spanish patients; however, the direction of the effect was similar. Due to lack of sufficient events in Spanish patients with GG-genotype, the analysis for OS and MD could not be performed (Table S11). We also observed other polymorphisms, rs10511694 (near gene-5 *IFNW1*), rs10081742 (7.20 kb from *5*′*IFNW1*) rs7038852 (near gene-3 *IFNA21*), rs632941, rs7043990 and rs7864960 were associated with one of the survival parameters (Table 2).

### Haplotype Analysis

Haplotypes were inferred for the polymorphisms genotyped located within a 26 kb region encompassing *IFNW1* and *IFNA21* on chromosome 9p22 that contained five SNPs that individually showed association with survival parameters. The analysis resulted in the inference of 40 haplotypes for the DFS and OS analysis and 31 haplotypes for the MD analysis for the polymorphisms rs10964859, rs105111694, rs2081381, rs10081742, rs10964862, rs10964863, rs10811482 and rs7038852. The most frequent haplotype taken as a reference was the one without any variant allele. GTCGACAA haplotype, found in 8.8% melanoma patients, showed an association with an increased risk MD (HR 1.94, 95%CI 1.16–3.26, P = 0.01) (Table 4). This haplotype included the minor alleles G and A of the rs10964859 and rs10964862 polymorphisms respectively, which were individually associated with an increased risk of MD. Other haplotypes were associated with worse prognosis in the DFS, OS and MD analysis; however, those occurred with a frequency ≤0.7 in the investigated patients (data not shown).

**Table 4:** MD haplotype analysis for the SNPs rs10964859 rs10511694 rs2081381 rs10081742 rs10964862 rs10964863 rs10811482 rs7038852 adjusted for the covariates age, gender and Breslow thickness.

haplotype	n	%	HR	CI	P
**C-C-C-A-C-C-A-A**	82	19.0	1.00	reference	–
**C-T-G-A-C-T-A-G**	70	16.2	1.42	(0.92–2.20)	0.11
**G-T-C-A-A-C-G-A**	60	13.9	1.49	(0.94–2.36)	0.09
**C-T-G-A-C-T-A-A**	51	11.8	1.05	(0.62–1.79)	0.86
**G-T-C-G-A-C-A-A**	38	8.8	1.94	(1.16–3.26)	**0.01**
**G-T-C-A-A-C-G-G**	37	8.6	1.38	(0.77–2.47)	0.28
**C-C-C-A-C-C-A-G**	23	5.3	0.90	(0.46–1.76)	0.77
**C-T-G-G-C-C-A-G**	10	2.3	1.07	(0.45–2.59)	0.87
**C-T-C-G-A-C-A-G**	9	2.1	1.48	(0.60–3.63)	0.39
**G-T-G-A-C-T-A-G**	7	1.6	1.45	(0.55–3.84)	0.45
**C-T-G-A-C-C-A-A**	6	1.4	1.55	(0.60–4.04)	0.37
**G-T-C-G-A-C-A-G**	6	1.4	1.09	(0.33–3.62)	0.88
**G-T-C-A-A-C-A-A**	4	0.9	0.38	(0.08–1.77)	0.22
**C-T-C-A-C-C-A-G**	3	0.7	0.48	(0.06–3.62)	0.47
**C-C-G-A-C-C-A-G**	3	0.7	0.35	(0.05–2.56)	0.30
**G-C-C-A-C-C-A-A**	3	0.7	19.56	(4.67–82.0)	**<.0001**
**C-T-G-A-C-C-A-G**	2	0.5	–	–	–
**C-T-G-G-C-T-A-G**	2	0.5	–	–	–
**C-C-C-A-C-T-A-A**	2	0.5	–	–	–
**C-C-G-A-C-T-A-G**	2	0.5	2.09	(0.49–8.99)	0.32
**G-T-C-A-A-T-A-A**	2	0.5	0.74	(0.10–5.54)	0.77
**C-T-C-A-C-C-G-G**	1	0.2	–	–	–
**C-T-C-G-A-C-G-A**	1	0.2	4.95	(0.61–39.8)	0.13
**C-T-G-A-A-C-G-A**	1	0.2	6.57	(0.85–51.0)	0.07
**C-T-G-G-C-C-A-A**	1	0.2	–	–	–
**C-C-C-A-C-C-G-G**	1	0.2	–	–	–
**C-C-C-A-C-T-A-G**	1	0.2	–	–	–
**C-C-C-G-A-C-A-G**	1	0.2	66.78	(7.29–612 )	**0.0002**
**C-C-G-A-C-C-A-A**	1	0.2	2.30	(0.29–18.6)	0.43
**G-T-C-A-C-C-G-A**	1	0.2	–	–	–
**G-T-G-G-A-T-A-G**	1	0.2	–	–	–

n = number of haplotypes in the population.

HR, Hazard Ratio; CI, Confidence Interval.

## Discussion

Polymorphisms in type I IFN genes have been reported to be associated with different diseases [17]–[21]. In this study we observed that, of all the type I *IFN* variants evaluated, two linked polymorphisms rs10964862 and rs10964859, located at 3′UTR of *IFNW1,* were associated with detrimental effects on survival of melanoma patients. Those associations with poor survival were confirmed in an independent population. One of the haplotypes within the region that contained variant alleles of the both rs10964859 and rs10964862 polymorphisms, was associated with an increased risk of death. The prediction analysis showed that the rs10964862 variant is contained in a sequence with a potential binding site for the GATA transcription factors family, GT-IIA, NF-1 and NP-TCII and the variant allele results in loss of those sites. The rs10964862 polymorphism has been previously reported to be associated with reduced tanning ability in melanoma-prone families [16].

The location of the cluster of type I *IFN* genes on chromosome 9p22 is in contiguity with a region associated with melanoma pathogenesis [30]. In addition to the known tumor suppressor genes *CDKN2A* and *CDKN2B*, it was suggested that other genes and loci in this region may also be involved in melanoma and cutaneous nevi development [31]–[33]. An earlier report showed location of deletion breakpoints within the *IFN* gene cluster in primary leukemia cells that resulted in partial loss of the *IFN* genes on the short arm of chromosome 9p. It was suggested that, in addition to the classic two-hit tumor suppressor gene model, the loss of the *IFN* genes, when it occurs, may play an additional role in the progression of these tumors [34]. Moreover, the possibility that genetic variants in the region 9p21 exert effects through altering the expression of *CDKN2A* and/or genes in the region in addition to their own functional relevance has also been suggested [16]. Variants associated with coronary artery disease, located in an enhancer interval on 9p21 locus, physically interact with *CDKN2A/B* and *MTAP* genes and with another interval downstream of *IFNA21*. Interestingly, this interval coincides with the region where we found the strongest associated variants with melanoma survival. In addition, it has been observed that the long-range interactions as well as the transcriptional regulation of the 9p21 locus were affected by IFN-γ treatment [35]. Based on our observations, we hypothesize that the region of 26 kb encompassing *IFNW1* and *IFNA21* could be a regulatory region in chromosome 9p22, containing natural genetic variants, which might play a role in disease outcome in melanoma. The variants rs10964859 and rs10964862 or others in linkage might be regulatory genetic variants and probably those have some influence on *IFNW1* gene through disruption of miRNA or transcription binding sites.

Melanoma therapy with IFNA has shown limited clinical efficacy, however remarkable survival response has been reported for a small group of patients, who also were predisposed to autoimmunity [36], [37]. It has been suggested that the genetic background of the individuals could be partly the reason for the observed variation in the treatment response [38]. Our data showed that variation in the effect of genotypes on the disease outcome was dependent on whether the patients were treated with IFNA.

In accordance with published reports, the beneficial effects of IFNA in the treatment of melanoma patients were observed at the beginning of the disease; however, with advancement of the disease, the effects of IFN treatment become less pronounced [39], [40]. IFN seems to be active in the eradication of micrometastasis and in the prevention of relapse. According to our data, melanoma patients who were carriers of the minor allele genotypes for rs10964859 and rs10964862 had an increased risk of metastasis, if they did not receive IFN therapy. However, their risk of death was augmented under treatment with IFN.

Our study shows that genetic variants in type I *IFN* genes play a role in melanoma disease and have an influence in the therapy outcome. Though it can be speculated, the effect being driven through variants in regulatory regions like 5′UTR or 3′UTR of *IFNW1* gene, the effect through some other functional linked SNP or gene beyond the region we studied cannot be ruled out. Polymorphisms in type I interferon genes that affect the biology of melanoma might be potentially used as predictors of survival and progression in the early stages of the disease. Genetic variants, which affect the efficacy of IFNA treatment, can also be useful for identification of patients for treatment. However, to achieve those levels of applications, it is imperative the findings reported in this study are confirmed further in large studies. It may, however, be pointed out that none of the associations between different polymorphisms and survival outcomes in melanoma patients remained significant after correction of for multiple hypothesis testing. Nevertheless, the observed associations between the rs10964862 and rs10964859 polymorphisms and survival parameters were uniform in two independent groups of patients.

## Supporting Information

Table S1 **Polymorphisms within interferon gene cluster selected for genotyping.**
(DOCX)

Table S2 **Detailed information about metastatic events in the German patients within and after the 10 years (10y) follow up.**
(DOCX)

Table S3 **Detailed information about death events in the German patients within and after the 10 years (10y) follow up.**
(DOCX)

Table S4 **Variation rs10964859 for the OS, DFS and MD analysis for the patients from Germany and Spain adjusted for the covariates age, gender and Breslow thickness.**
(DOCX)

Table S5 **Estimated 10 years DFS, OS and MD survival analysis for the group of patients from Germany “with only IFN” (** **Figure 2** **D) and “without only IFN” (** **Figure 2** **E) for the SNP rs10964859.**
(DOCX)

Table S6 **Estimated 10 years OS, DFS and MD survival analysis for the group of patients from Germany “with IFN” (** **Figure 2** **B) and “without IFN” (** **Figure 2** **C) for the SNP rs10964859.**
(DOCX)

Table S7 **Estimated 10 years OS, DFS and MD survival analysis for the group of patients from Spain “with only IFN” and “without only IFN” for the SNP rs10964859.**
(DOCX)

Table S8 **Estimated 10 years OS, DFS and MD survival analysis for the group of patients from Germany “with IFN” (** **Figure 2** **B) and “without IFN” (** **Figure 2** **C) for the SNP rs10964862.**
(DOCX)

Table S9 **Estimated 10 years OS, DFS and MD survival analysis for the group of patients from Germany “with only IFN” (** **Figure 2** **D) and “without only IFN” (** **Figure 2** **E) for the SNP rs10964862.**
(DOCX)

Table S10 **Estimated 10 years OS, DFS and MD survival analysis for the group of patients from Spain “with only IFN” and “without only IFN” for the SNP rs10964862.**
(DOCX)

Table S11 **Variation rs597408 for the OS, DFS and MD analysis for the patients from Germany and Spain adjusted for the covariates age, gender and Breslow thickness.**
(DOCX)

Table S12 **Estimated 10 years OS, DFS and MD survival analysis for the group of patients from Germany “with only IFN” (** **Figure 2** **D) and “without only IFN” (** **Figure 2** **E) for the SNP rs597408.**
(DOCX)
