# Supplementary material for: Influence of Genetic Variants in Type I Interferon Genes on Melanoma Survival and Therapy
Source: PLoS One. 2012 Nov 27;7(11):e50692. doi: 10.1371/journal.pone.0050692 (PMC3507747; doi:10.1371/journal.pone.0050692)
Supplement: Table S1 — Polymorphisms within interferon gene cluster selected for genotyping. (DOCX) [file pone.0050692.s001.docx]

| **contig location**  **Table S1. Polymorphisms within interferon gene cluster selected for genotyping** | **SNPs genotyped** | **Gene** | **number of SNPs tagged** |
| --- | --- | --- | --- |
| **21063945** | **rs1424860** |  | from GWAS |
| **21130672** | **rs10964859** | UTR-3 IFNW1 |  |
| **21133020** | **rs10511694** | nearGene-5 IFNW1 | 6 |
| **21138599** | **rs2081381** |  | 2 |
| **21139391** | **rs10081742** |  |  |
| **21141553** | **rs10964862** |  |  |
| **21143106** | **rs10964863** |  | 1 |
| **21143985** | **rs10811482** |  | 1 |
| **21155322** | **rs7038852** | nearGene-3 IFNA21 | 9 |
| **21174475** | **rs4568676** |  | 10 |
| **21177700** | **rs2383183** | nearGene-5 IFNA4 |  |
| **21197037** | **rs10119910** | STOP-GAIN IFNA10 | from literature [100] |
| **21208096** | **rs10964912** | nearGene-5 IFNA16 | 1 |
| **21210121** | **rs13340713** |  |  |
| **21245150** | **rs1330320** |  | 17 |
| **21294803** | **rs10757212** | coding-synonymous IFNA5 | 44 |
| **21296318** | **rs7031048** | nearGene-5 IFNA5 | 19 |
| **21297784** | **rs3758236** |  | 75 |
| **21317141** | **rs913931** |  |  |
| **21351957** | **rs597408** |  |  |
| **21368325** | **rs10448208** |  | 3 |
| **21371286** | **rs647167** |  | 8 |
| **21373734** | **rs615544** |  |  |
| **21374363** | **rs10120977** | UTR-3 IFNA2 | 11 |
| **21378712** | **rs632941** |  | 8 |
| **21381698** | **rs1224391** |  | 1 |
| **21391106** | **rs4978113** |  | 4 |
| **21397250** | **rs1330322** | nearGene-5 IFNA8 | 3 |
| **21405687** | **rs7025006** |  | 1 |
| **21409723** | **rs2104880** |  | 1 |
| **21410677** | **rs1332179** |  | 1 |
| **21413520** | **rs1591032** |  |  |
| **21415083** | **rs7871767** |  |  |
| **21418770** | **rs7043990** |  | 1 |
| **21420131** | **rs12337364** |  | 2 |
| **21428684** | **rs1332190** | nearGene-5 IFNA1 |  |
| **21438448** | **rs7864960** |  |  |
| **21440474** | **rs6475535** |  | 3 |
| **21446776** | **rs10491569** |  |  |
| **21457712** | **rs1888888** |  | 1 |
| **21461499** | **rs1412395** |  | 2 |
| **21461726** | **rs2383192** |  | 1 |
| **21471556** | **rs1125488** | missense IFNE | 18 |
| **21520609** | **rs10811561** |  | from GWAS |
