# Supplementary material for: Influence of Genetic Variants in Type I Interferon Genes on Melanoma Survival and Therapy
Source: PLoS One. 2012 Nov 27;7(11):e50692. doi: 10.1371/journal.pone.0050692 (PMC3507747; doi:10.1371/journal.pone.0050692)
Supplement: Table S2 — Detailed information about metastatic events in the German patients within and after the 10 years (10y) follow up. (DOCX) [file pone.0050692.s002.docx]

**Table S2. Detailed information about metastatic events in the German patients within and after the 10 years (10y) follow up**

|  | **GERMANY** | | | | | | | **SPAIN** | | | | | | |
| --- | --- | --- | --- | --- | --- | --- | --- | --- | --- | --- | --- | --- | --- | --- |
| **All patients with skin melanoma** | **n** | **TM** | **%** | **Mw10y** | **%** | **Ma10y** | **%** | **n** | **TM** | **%** | **Mw10y** | **%** | **Ma10y** | **%** |
| **first diagnosis all stages** | 752 | 379 | 50 | 319 | 42 | 60 | 8 | 837 | 146 | 17 | 110 | 13 | 36 | 4 |
| **last contact within 10y** | 563 | 211 | 38 | 211 | 38 | - | - | 589 | 64 | 11 | 64 | 11 | - | 0 |
| **last contact after 10y** | 58 | 41 | 71 | - | - | 41 | 71 | 193 | 31 | 16 | - | - | 31 | 16 |
| **without last contact information** | 131 | 127 | 97 | 108 | 82 | 19 | 15 | 55 | 51 | 93 | 46 | 84 | 5 | 9 |
| **Patients with AJCC stage 0, I or II at first diagnosis** | **n** | **TM** | **%** | **Mw10y** | **%** | **Ma10y** | **%** | **n** | **TM** | **%** | **Mw10y** | **%** | **Ma10y** | **%** |
| **first diagnosis stage 0/I/II** | 625 | 257 | 41 | 200 | 32 | 57 | 9.1 | 710 | 92 | 13 | 63 | 9 | 29 | 4 |
| **last contact within 10y** | 476 | 128 | 27 | 128 | 27 | - | - | 504 | 35 | 7 | 35 | 7 | - | 0 |
| **last contact after 10y** | 55 | 38 | 69 | - | - | 38 | 69 | 172 | 24 | 14 | - | - | 24 | 14 |
| **without last contact information** | 94 | 91 | 97 | 72 | 77 | 19 | 20 | 34 | 33 | 97 | 28 | 82 | 5 | 15 |
| **Patients with AJCC stage 0, I or II at FD and complete information for Age, Gender and Breslow thickness** | **n** | **TM** | **%** | **Mw10y** | **%** | **Ma10y** | **%** | **n** | **TM** | **%** | **Mw10y** | **%** | **Ma10y** | **%** |
| **first diagnosis stage 0/I/II** | 541 | 218 | 40 | 173 | 32 | 45 | 8.3 | 638 | 87 | 14 | 63 | 10 | 24 | 4 |
| **last contact within 10y** | 416 | 110 | 26 | 110 | 26 | - | - | 450 | 35 | 8 | 35 | 8 | - | 0 |
| **last contact after 10y** | 47 | 30 | 64 | - | - | 30 | 64 | 154 | 19 | 12 | - | - | 19 | 12 |
| **without last contact information** | 78 | 78 | 100 | 63 | 81 | 15 | 19 | 34 | 33 | 97 | 28 | 82 | 5 | 15 |

n, number of patients; TM, Total Metastasis; Mw10y, Metastasis within 10 years; Ma10y, Metastasis after 10 years
