# Supplementary material for: Influence of Genetic Variants in Type I Interferon Genes on Melanoma Survival and Therapy
Source: PLoS One. 2012 Nov 27;7(11):e50692. doi: 10.1371/journal.pone.0050692 (PMC3507747; doi:10.1371/journal.pone.0050692)
Supplement: Table S3 — Detailed information about death events in the German patients within and after the 10 years (10y) follow up. (DOCX) [file pone.0050692.s003.docx]

**Table S3. Detailed information about death events in the German patients within and after the 10 years (10y) follow up**

|  | **GERMANY** | | | | | | | **SPAIN** | | | | | | |
| --- | --- | --- | --- | --- | --- | --- | --- | --- | --- | --- | --- | --- | --- | --- |
| **All patients with skin melanoma** | **n** | **TD** | **%** | **Dw10y** | **%** | **Da10y** | **%** | **n** | **TD** | **%** | **Dw10y** | **%** | **Da10y** | **%** |
| **first diagnosis all stages** | 752 | 238 | 32 | 207 | 28 | 31 | 4.1 | 837 | 77 | 9 | 72 | 9 | 5 | 1 |
| **last contact within 10y** | 563 | 95 | 17 | 95 | 17 | - | - | 589 | 22 | 4 | 22 | 4 | - | - |
| **last contact after 10y** | 58 | 12 | 21 | - | - | 12 | 21 | 193 | - | - | - | - | - | - |
| **without last contact information** | 131 | 131 | 100 | 112 | 86 | 19 | 15 | 55 | 55 | 100 | 50 | 91 | 5 | 9 |
| **Patients with AJCC stage 0, I or II at first diagnosis** | **n** | **TD** | **%** | **Dw10y** | **%** | **Da10y** | **%** | **n** | **TD** | **%** | **Dw10y** | **%** | **Da10y** | **%** |
| **First diagnosis stage 0/I/II** | 625 | 174 | 28 | 143 | 23 | 31 | 5 | 710 | 45 | 6 | 40 | 6 | 5 | 1 |
| **last contact within 10y** | 476 | 68 | 12 | 68 | 14 | - | - | 504 | 11 | 2 | 11 | 2 | - | - |
| **last contact after 10y** | 55 | 12 | 22 | - | - | 12 | 22 | 172 | - | - | - | - | - | - |
| **without last contact information** | 94 | 94 | 100 | 75 | 80 | 19 | 20 | 34 | 34 | 100 | 29 | 85 | 5 | 15 |
| **Patients with AJCC stage 0, I or II at FD and complete information for Age, Gender and Breslow thickness** | **n** | **TD** | **%** | **Dw10y** | **%** | **Da10y** | **%** | **n** | **TD** | **%** | **Dw10y** | **%** | **Da10y** | **%** |
| **First diagnosis stage 0/I/II** | 541 | 146 | 27 | 122 | 23 | 24 | 4.4 | 638 | 45 | 7 | 40 | 6 | 5 | 1 |
| **last contact within 10y** | 416 | 59 | 14 | 59 | 14 | - | - | 450 | 11 | 2 | 11 | 2 | - | - |
| **last contact after 10y** | 47 | 9 | 19 | - | - | 9 | 19 | 154 | - | - | - | - | - | - |
| **without last contact information** | 78 | 78 | 100 | 63 | 81 | 15 | 19 | 34 | 34 | 100 | 29 | 85 | 5 | 15 |

n, number of patients; TD, Total Deaths; Dw10y, Deaths within 10 years; Da10y, Deaths after 10 years
