# Supplementary material for: Influence of Genetic Variants in Type I Interferon Genes on Melanoma Survival and Therapy
Source: PLoS One. 2012 Nov 27;7(11):e50692. doi: 10.1371/journal.pone.0050692 (PMC3507747; doi:10.1371/journal.pone.0050692)
Supplement: Table S4 — Variation rs10964859 for the OS, DFS and MD analysis for the patients from Germany and Spain adjusted for the covariates age, gender and Breslow thickness. (DOCX) [file pone.0050692.s004.docx]

**Table S4. Variation rs10964859 for the OS, DFS and MD analysis for the patients from Germany and Spain adjusted for the covariates age, gender and Breslow thickness**

| rs10964859 | genotype | cases | n | % | HR | CI | P |
| --- | --- | --- | --- | --- | --- | --- | --- |
| OS GERMAN | CC | 232 | 43 | 18.5 | 1.00 | (referent) | - |
|  | CG | 247 | 57 | 23.1 | 1.16 | (0.77 - 1.73) | 0.48 |
|  | GG | 57 | 17 | 29.8 | 1.80 | (1.02 - 3.16) | **0.04** |
|  | CG +GG | 304 | 74 | 24.3 | 1.27 | (0.87 - 1.86) | 0.22 |
| OS SPANISH | CC | 262 | 11 | 4.2 | 1.00 | (referent) | - |
|  | CG | 291 | 22 | 7.6 | 1.71 | (0.83 - 3.54) | 0.15 |
|  | GG | 73 | 5 | 6.8 | 1.66 | (0.57 - 4.79) | 0.35 |
|  | CG +GG | 364 | 27 | 7.4 | 1.7 | (0.84 - 3.44) | 0.14 |
| DFS GERMAN | CC | 232 | 72 | 31.0 | 1.00 | (referent) | - |
|  | CG | 247 | 97 | 39.3 | 1.29 | (0.94 - 1.76) | 0.11 |
|  | GG | 57 | 26 | 45.6 | 1.51 | (0.96 - 2.37) | 0.07 |
|  | CG +GG | 304 | 123 | 40.5 | 1.33 | (0.99 - 1.79) | 0.06 |
| DFS SPANISH | CC | 262 | 26 | 9.9 | 1.00 | (referent) | - |
|  | CG | 291 | 40 | 13.7 | 1.36 | (0.83 - 2.24) | 0.22 |
|  | GG | 73 | 8 | 11.0 | 1.20 | (0.54 - 2.66) | 0.65 |
|  | CG +GG | 364 | 48 | 13.2 | 1.33 | (0.83 - 2.15) | 0.24 |
| MD GERMAN | CC | 84 | 51 | 60.7 | 1.00 | (referent) | - |
|  | CG | 104 | 62 | 59.6 | 1.00 | (0.68 - 1.46) | 0.98 |
|  | GG | 27 | 20 | 74.1 | 2.01 | (1.17 - 3.44) | **0.01** |
|  | CG +GG | 131 | 82 | 62.6 | 1.14 | (0.80 - 1.63) | 0.46 |
| MD SPANISH | CC | 29 | 14 | 48.3 | 1.00 | (referent) | - |
|  | CG | 46 | 22 | 47.8 | 1.14 | (0.58 - 2.27) | 0.70 |
|  | GG | 9 | 5 | 55.6 | 1.44 | (0.51 - 4.04) | 0.49 |
|  | CG +GG | 55 | 27 | 49.1 | 1.19 | (0.61 - 2.30) | 0.61 |

n, number of deaths for OS and MD analysis or number of metastases for DFS analysis

OS, overall survival; DFS, disease free progression; MD, metastasis to death

HR, Hazard Ratio; CI, Confidence Interval
