# Supplementary material for: Influence of Genetic Variants in Type I Interferon Genes on Melanoma Survival and Therapy
Source: PLoS One. 2012 Nov 27;7(11):e50692. doi: 10.1371/journal.pone.0050692 (PMC3507747; doi:10.1371/journal.pone.0050692)
Supplement: Table S5 — Estimated 10 years DFS, OS and MD survival analysis for the group of patients from Germany “with only IFN” ( Figure 2 D) and “without only IFN” ( Figure 2 E) for the SNP rs10964859. (DOCX) [file pone.0050692.s005.docx]

**Table S5. Estimated 10 years DFP, OS and MD survival analysis for the group of patients from Germany “with only IFN” (Figure 2 D) and “without only IFN” (Figure 2 E) for the SNP rs10964859**

| 84 patients from Germany “WITH ONLY IFN” ^a^ | | | | | | | |
| --- | --- | --- | --- | --- | --- | --- | --- |
| rs10964859 | **genotype** | **cases** | **n** | **%** | **HR^§^** | **CI^§^** | **P^§^** |
| OS | CC | 36 | 4 | 11.1 | 1.00 | (referent) | - |
|  | CG | 39 | 9 | 23.1 | 2.64 | (0.77 - 9.00) | 0.12 |
|  | GG | 7 | - | - | - | - | - |
|  | CG +GG | 46 | 9 | 19.6 | 2.14 | (0.63 - 7.24) | 0.22 |
| DFP | CC | 36 | 15 | 41.7 | 1.00 | (referent) | - |
|  | CG | 39 | 17 | 43.6 | 1.45 | (0.69 - 3.05) | 0.33 |
|  | GG | 7 | 3 | 42.9 | 1.06 | (0.30 - 3.69) | 0.93 |
|  | CG +GG | 46 | 20 | 43.5 | 1.36 | (0.67 - 2.76) | 0.39 |
| MD | CC | 17 | 4 | 23.5 | 1.00 | (referent) | - |
|  | CG | 17 | 9 | 52.9 | 3.55 | (1.01 - 12.4) | **0.05** |
|  | GG | 4 | - | - | - | - | - |
|  | CG +GG | 21 | 9 | 42.9 | 2.81 | (0.82 - 9.63) | 0.10 |
| 457 patients from Germany “WITHOUT ONLY IFN” ^b^ | | | | | | | |
| rs10964859 | **genotype** | **cases** | **n** | **%** | **HR*** | **CI*** | **P*** |
| OS | CC | 196 | 39 | 19.9 | 1.00 | (referent) | - |
|  | CG | 207 | 48 | 23.2 | 1.05 | (0.67 - 1.62) | 0.84 |
|  | GG | 50 | 17 | 34.0 | 1.84 | (1.04 - 3.26) | **0.04** |
|  | CG +GG | 257 | 65 | 25.3 | 1.20 | (0.80 - 1.80) | 0.39 |
| DFP | CC | 196 | 57 | 29.1 | 1.00 | (referent) | - |
|  | CG | 207 | 79 | 38.2 | 1.28 | (0.90 - 1.82) | 0.17 |
|  | GG | 50 | 23 | 46.0 | 1.61 | (0.99 - 2.62) | **0.05** |
|  | CG +GG | 257 | 102 | 39.7 | 1.35 | (0.97 - 1.88) | 0.08 |
| MD | CC | 67 | 47 | 70.1 | 1.00 | (referent) | - |
|  | CG | 86 | 53 | 61.6 | 0.82 | (0.55 - 1.23) | 0.35 |
|  | GG | 23 | 20 | 87.0 | 1.80 | (1.04 - 3.11) | **0.04** |
|  | CG +GG | 109 | 73 | 67.0 | 0.98 | (0.67 - 1.42) | 0.90 |

**^a^** only IFN as treatment

**^b^** no therapy or different kinds of therapies combined or not with IFN

n number of deaths for OS and MD analysis or number of metastasis for DFP analysis

**^§^** adjusted for age, gender, Breslow thickness and treatment as time-dependent variable

*adjusted for age, gender and Breslow thickness

HR, Hazard Ratio; CI, Confidence Interval
