# Supplementary material for: Influence of Genetic Variants in Type I Interferon Genes on Melanoma Survival and Therapy
Source: PLoS One. 2012 Nov 27;7(11):e50692. doi: 10.1371/journal.pone.0050692 (PMC3507747; doi:10.1371/journal.pone.0050692)
Supplement: Table S6 — Estimated 10 years OS, DFS and MD survival analysis for the group of patients from Germany “with IFN” ( Figure 2 B) and “without IFN” ( Figure 2 C) for the SNP rs10964859. (DOCX) [file pone.0050692.s006.docx]

**Table S6. Estimated 10 years OS, DFP and MD survival analysis for the group of patients from Germany “with IFN” (Figure 2 B) and “without IFN” (Figure 2 C) for the SNP rs10964859**

| 148 patients from Germany “WITH IFN” ^a^ | | | | | | | |
| --- | --- | --- | --- | --- | --- | --- | --- |
| rs10964859 | **genotype** | **cases** | **n** | **%** | **HR*** | **CI*** | **P*** |
| OS | CC | 56 | 17 | 30.4 | 1.00 | (referent) | - |
|  | CG | 68 | 25 | 36.8 | 1.20 | (0.64 - 2.27) | 0.57 |
|  | GG | 21 | 12 | 57.1 | 2.38 | (1.13 - 5.02) | **0.02** |
|  | CG +GG | 89 | 37 | 41.6 | 1.46 | (0.81 - 2.63) | 0.21 |
| DFP | CC | 56 | 32 | 57.1 | 1.00 | (referent) | - |
|  | CG | 68 | 44 | 64.7 | 1.35 | (0.84 - 2.15) | 0.21 |
|  | GG | 21 | 17 | 81.0 | 1.51 | (0.84 - 2.73) | 0.17 |
|  | CG +GG | 89 | 61 | 68.5 | 1.39 | (0.90 - 2.15) | 0.14 |
| MD | CC | 36 | 19 | 52.8 | 1.00 | (referent) | - |
|  | CG | 46 | 29 | 63.0 | 1.18 | (0.65 - 2.13) | 0.59 |
|  | GG | 18 | 14 | 77.8 | 3.08 | (1.48 - 6.40) | **0.003** |
|  | CG +GG | 64 | 43 | 67.2 | 1.48 | (0.86 - 2.57) | 0.16 |
| 393 patients from Germany “WITHOUT IFN” ^b^ | | | | | | | |
| rs10964859 | **genotype** | **cases** | **n** | **%** | **HR*** | **CI*** | **P*** |
| OS | CC | 176 | 26 | 14.8 | 1.00 | (referent) | - |
|  | CG | 178 | 32 | 18.0 | 1.12 | (0.66 - 1.90) | 0.67 |
|  | GG | 36 | 5 | 13.9 | 1.09 | (0.42 - 2.86) | 0.86 |
|  | CG +GG | 214 | 37 | 17.3 | 1.12 | (0.67 - 1.86) | 0.67 |
| DFP | CC | 176 | 40 | 22.7 | 1.00 | (referent) | - |
|  | CG | 178 | 52 | 29.2 | 1.26 | (0.83 - 1.91) | 0.28 |
|  | GG | 36 | 9 | 25.0 | 1.16 | (0.56 - 2.41) | 0.69 |
|  | CG +GG | 214 | 61 | 28.5 | 1.24 | (0.83 - 1.86) | 0.29 |
| MD | CC | 48 | 32 | 66.7 | 1.00 | (referent) | - |
|  | CG | 57 | 33 | 57.9 | 0.88 | (0.53 - 1.46) | 0.62 |
|  | GG | 9 | 6 | 66.7 | 1.34 | (0.55 - 3.30) | 0.52 |
|  | CG +GG | 66 | 39 | 59.1 | 0.94 | (0.58 - 1.51) | 0.79 |

^a^ IFN alone or combined with other treatments

^b^ no treatment or different kinds of therapies but never IFN

n number of deaths for OS and MD analysis or number of metastasis for DFP analysis

*adjusted for age, gender and Breslow thickness

HR, Hazard Ratio; CI, Confidence Interval
