# Supplementary material for: Influence of Genetic Variants in Type I Interferon Genes on Melanoma Survival and Therapy
Source: PLoS One. 2012 Nov 27;7(11):e50692. doi: 10.1371/journal.pone.0050692 (PMC3507747; doi:10.1371/journal.pone.0050692)
Supplement: Table S7 — Estimated 10 years OS, DFS and MD survival analysis for the group of patients from Spain “with only IFN” and “without only IFN” for the SNP rs10964859. (DOCX) [file pone.0050692.s007.docx]

**Table S7. Estimated 10 years OS, DFP and MD survival analysis for the group of patients from Spain “with only IFN” and “without only IFN” for the SNP rs10964859**

| 142 patients from Spain “WITH ONLY IFN” ^a^ | | | | | | | |
| --- | --- | --- | --- | --- | --- | --- | --- |
| rs10964859 | **genotype** | **cases** | **n** | **%** | **HR^§^** | **CI^§^** | **P^§^** |
| OS | CC | 70 | 4 | 5.7 | 1.00 | (referent) | - |
|  | CG | 55 | 7 | 12.7 | 3.27 | (0.87 - 12.3) | 0.08 |
|  | GG | 17 | 2 | 11.8 | 3.39 | (0.53 - 21.8) | 0.20 |
|  | CG +GG | 72 | 9 | 12.5 | 3.29 | (0.90 - 12.0) | 0.07 |
| DFP | CC | 70 | 14 | 20.0 | 1.00 | (referent) | - |
|  | CG | 55 | 10 | 18.2 | 0.92 | (0.40 - 2.09) | 0.84 |
|  | GG | 17 | 3 | 17.6 | 0.83 | (0.23 - 2.97) | 0.77 |
|  | CG +GG | 72 | 13 | 18.0 | 0.90 | (0.42 - 1.94) | 0.78 |
| MD | CC | 15 | 4 | 26.7 | 1.00 | (referent) | - |
|  | CG | 11 | 6 | 54.5 | 2.75 | (0.59 - 12.9) | 0.20 |
|  | GG | 4 | 2 | 50.0 | 3.74 | (0.57 - 24.4) | 0.17 |
|  | CG +GG | 15 | 8 | 53.3 | 3.06 | (0.78 - 12.0) | 0.11 |
| 496 patients from Spain “WITHOUT ONLY IFN” ^b^ | | | | | | | |
| rs10964859 | **genotype** | **cases** | **n** | **%** | **HR*** | **CI*** | **P*** |
| OS | CC | 192 | 7 | 3.6 | 1.00 | (referent) | - |
|  | CG | 236 | 15 | 6.4 | 1.65 | (0.67 - 4.06) | 0.28 |
|  | GG | 56 | 3 | 5.4 | 1.60 | (0.41 - 6.21) | 0.50 |
|  | CG +GG | 292 | 18 | 6.2 | 1.64 | (0.68 - 3.94) | 0.27 |
| DFP | CC | 192 | 12 | 6.3 | 1.00 | (referent) | - |
|  | CG | 236 | 30 | 12.8 | 1.92 | (0.97 - 3.78) | 0.06 |
|  | GG | 56 | 5 | 8.9 | 1.70 | (0.60 - 4.83) | 0.32 |
|  | CG +GG | 292 | 35 | 12.0 | 1.88 | (0.97 - 3.65) | 0.06 |
| MD | CC | 14 | 10 | 71.4 | 1.00 | (referent) | - |
|  | CG | 35 | 16 | 45.7 | 0.54 | (0.22 - 1.32) | 0.18 |
|  | GG | 5 | 3 | 60.0 | 0.47 | (0.12 - 1.85) | 0.28 |
|  | CG +GG | 40 | 19 | 47.5 | 0.53 | (0.22 - 1.26) | 0.15 |

**^a^** only IFN as therapy

**^b^** no treatment

n number of deaths for OS and MD analysis or number of metastasis for DFP analysis

**^§^** adjusted for age, gender, Breslow thickness and treatment as time-dependent variable

*adjusted for age, gender and Breslow thickness

HR, Hazard Ratio; CI, Confidence Interval
