# Supplementary material for: Influence of Genetic Variants in Type I Interferon Genes on Melanoma Survival and Therapy
Source: PLoS One. 2012 Nov 27;7(11):e50692. doi: 10.1371/journal.pone.0050692 (PMC3507747; doi:10.1371/journal.pone.0050692)
Supplement: Table S8 — Estimated 10 years OS, DFS and MD survival analysis for the group of patients from Germany “with IFN” ( Figure 2 B) and “without IFN” ( Figure 2 C) for the SNP rs10964862. (DOCX) [file pone.0050692.s008.docx]

**Table S8. Estimated 10 years OS, DFP and MD survival analysis for the group of patients from Germany “with IFN” (Figure 2 B) and “without IFN” (Figure 2 C) for the SNP rs10964862**

| 148 patients from Germany “WITH IFN” ^a^ | | | | | | | |
| --- | --- | --- | --- | --- | --- | --- | --- |
| rs10964862 | **genotype** | **cases** | **n** | **%** | **HR*** | **CI*** | **P*** |
| OS | CC | 49 | 15 | 30.6 | 1.00 | (referent) | - |
|  | CA | 78 | 30 | 38.5 | 1.40 | (0.74 - 2.65) | 0.30 |
|  | AA | 19 | 9 | 47.4 | 1.91 | (0.83 - 4.40) | 0.13 |
|  | CA +AA | 97 | 39 | 40.2 | 1.50 | (0.82 - 2.76) | 0.19 |
| DFP | CC | 49 | 29 | 59.2 | 1.00 | (referent) | - |
|  | CA | 78 | 51 | 65.4 | 1.32 | (0.82 - 2.11) | 0.25 |
|  | AA | 19 | 14 | 73.7 | 1.29 | (0.68 - 2.45) | 0.43 |
|  | CA +AA | 97 | 65 | 67.0 | 1.31 | (0.84 - 2.05) | 0.23 |
| MD | CC | 33 | 18 | 54.5 | 1.00 | (referent) | - |
|  | CA | 53 | 33 | 62.3 | 1.30 | (0.73 - 2.33) | 0.38 |
|  | AA | 15 | 11 | 73.3 | 2.62 | (1.19 - 5.73) | **0.02** |
|  | CA +AA | 68 | 44 | 64.7 | 1.48 | (0.85 - 2.59) | 0.17 |
| 393 patients from Germany “WITHOUT IFN” ^b^ | | | | | | | |
| rs10964862 | **genotype** | **cases** | **n** | **%** | **HR*** | **CI*** | **P*** |
| OS | CC | 182 | 28 | 15.4 | 1.00 | (referent) | - |
|  | CA | 174 | 30 | 17.2 | 0.93 | (0.55 - 1.60) | 0.80 |
|  | AA | 30 | 5 | 16.7 | 1.22 | (0.46 - 3.20) | 0.69 |
|  | CA +AA | 204 | 35 | 17.2 | 0.97 | (0.58 - 1.62) | 0.90 |
| DFP | CC | 182 | 40 | 22.0 | 1.00 | (referent) | - |
|  | CA | 174 | 52 | 29.9 | 1.52 | (1.00 - 2.30) | **0.05** |
|  | AA | 30 | 9 | 30.0 | 1.57 | (0.75 - 3.28) | 0.23 |
|  | CA +AA | 204 | 61 | 29.9 | 1.52 | (1.02 - 2.28) | **0.04** |
| MD | CC | 47 | 29 | 61.7 | 1.00 | (referent) | - |
|  | CA | 58 | 36 | 62.1 | 0.87 | (0.52 - 1.46) | 0.60 |
|  | AA | 9 | 6 | 66.7 | 1.31 | (0.53 - 3.29) | 0.56 |
|  | CA +AA | 67 | 42 | 62.7 | 0.92 | (0.56 - 1.51) | 0.74 |

^a^ IFN alone or combined with other treatments

^b^ no treatment or different kinds of therapies but never IFN

n number of deaths for OS and MD analysis or number of metastasis for DFP analysis

*adjusted for age, gender and Breslow thickness

HR, Hazard Ratio; CI, Confidence Interval
