# Supplementary material for: Influence of Genetic Variants in Type I Interferon Genes on Melanoma Survival and Therapy
Source: PLoS One. 2012 Nov 27;7(11):e50692. doi: 10.1371/journal.pone.0050692 (PMC3507747; doi:10.1371/journal.pone.0050692)
Supplement: Table S9 — Estimated 10 years OS, DFS and MD survival analysis for the group of patients from Germany “with only IFN” ( Figure 2 D) and “without only IFN” ( Figure 2 E) for the SNP rs10964862. (DOCX) [file pone.0050692.s009.docx]

**Table S9. Estimated 10 years OS, DFP and MD survival analysis for the group of patients from Germany “with only IFN” (Figure 2 D) and “without only IFN” (Figure 2 E) for the SNP rs10964862**

| 84 patients from Germany “WITH ONLY IFN” ^a^ | | | | | | | |
| --- | --- | --- | --- | --- | --- | --- | --- |
| rs10964862 | **genotype** | **cases** | **n** | **%** | **HR^§^** | **CI^§^** | **P^§^** |
| OS | CC | 31 | 4 | 12.9 | 1.00 | (referent) | - |
|  | CA | 44 | 9 | 20.5 | 2.13 | (0.62 - 7.33) | 0.23 |
|  | AA | 8 | - | - | - | - | - |
|  | CA +AA | 52 | 9 | 17.3 | 1.77 | (0.52 - 6.04) | 0.36 |
| DFP | CC | 31 | 13 | 41.9 | 1.00 | (referent) | - |
|  | CA | 44 | 20 | 45.5 | 1.41 | (0.67 - 2.96) | 0.36 |
|  | AA | 8 | 3 | 37.5 | 1.02 | (0.29 - 3.62) | 0.98 |
|  | CA +AA | 52 | 23 | 44.2 | 1.33 | (0.65 - 2.72) | 0.43 |
| MD | CC | 15 | 4 | 26.7 | 1.00 | (referent) | - |
|  | CA | 20 | 9 | 45.0 | 2.48 | (0.70 - 8.73) | 0.16 |
|  | AA | 4 | - | - | - | - | - |
|  | CA +AA | 24 | 9 | 37.5 | 2.02 | (0.59 - 6.98) | 0.27 |
| 457 patients from Germany “WITHOUT ONLY IFN” ^b^ | | | | | | | |
| rs10964862 | **genotype** | **cases** | **n** | **%** | **HR*** | **CI*** | **P*** |
| OS | CC | 200 | 39 | 19.5 | 1.00 | (referent) | - |
|  | CA | 208 | 51 | 24.5 | 1.06 | (0.69 - 1.64) | 0.79 |
|  | AA | 41 | 14 | 34.1 | 1.74 | (0.94 - 3.23) | 0.08 |
|  | CA +AA | 249 | 65 | 26.1 | 1.17 | (0.78 - 1.76) | 0.45 |
| DFP | CC | 200 | 56 | 28.0 | 1.00 | (referent) | - |
|  | CA | 208 | 83 | 39.9 | 1.42 | (1.00 - 2.00) | **0.05** |
|  | AA | 41 | 20 | 48.8 | 1.78 | (1.06 - 2.98) | **0.03** |
|  | CA +AA | 249 | 103 | 41.4 | 1.48 | (1.06 - 2.06) | **0.02** |
| MD | CC | 65 | 43 | 66.2 | 1.00 | (referent) | - |
|  | CA | 91 | 60 | 65.9 | 0.87 | (0.58 - 1.31) | 0.51 |
|  | AA | 20 | 17 | 85.0 | 1.66 | (0.92 - 2.99) | 0.09 |
|  | CA +AA | 111 | 77 | 69.4 | 0.97 | (0.66 - 1.43) | 0.88 |

**^a^** only IFN as treatment

**^b^** no therapy or different kinds of therapies combined or not with IFN

n number of deaths for OS and MD analysis or number of metastasis for DFP analysis

**^§^** adjusted for age, gender, Breslow thickness and treatment as time-dependent variable

*adjusted for age, gender and Breslow thickness

HR, Hazard Ratio; CI, Confidence Interval
