# Supplementary material for: Influence of Genetic Variants in Type I Interferon Genes on Melanoma Survival and Therapy
Source: PLoS One. 2012 Nov 27;7(11):e50692. doi: 10.1371/journal.pone.0050692 (PMC3507747; doi:10.1371/journal.pone.0050692)
Supplement: Table S10 — Estimated 10 years OS, DFS and MD survival analysis for the group of patients from Spain “with only IFN” and “without only IFN” for the SNP rs10964862. (DOCX) [file pone.0050692.s010.docx]

**Table S10. Estimated 10 years OS, DFP and MD survival analysis for the group of patients from Spain “with only IFN” and “without only IFN” for the SNP rs10964862**

| 142 patients from Spain “WITH ONLY IFN” ^a^ | | | | | | | |
| --- | --- | --- | --- | --- | --- | --- | --- |
| rs10964862 | **genotype** | **cases** | **n** | **%** | **HR^§^** | **CI^§^** | **P^§^** |
| OS | CC | 66 | 4 | 6.1 | 1.00 | (referent) | - |
|  | CA | 56 | 7 | 12.5 | 3.21 | (0.85 - 12.1) | 0.09 |
|  | AA | 20 | 2 | 10.0 | 2.90 | (0.47 - 17.9) | 0.25 |
|  | CA +AA | 76 | 9 | 11.8 | 3.15 | (0.86 - 11.5) | 0.08 |
| DFP | CC | 66 | 12 | 18.2 | 1.00 | (referent) | - |
|  | CA | 56 | 12 | 21.4 | 1.24 | (0.55 - 2.80) | 0.61 |
|  | AA | 20 | 3 | 15.0 | 0.82 | (0.23 - 2.95) | 0.76 |
|  | CA +AA | 76 | 15 | 19.7 | 1.12 | (0.52 - 2.44) | 0.77 |
| MD | CC | 13 | 4 | 30.8 | 1.00 | (referent) | - |
|  | CA | 13 | 6 | 46.2 | 1.96 | (0.44 - 8.81) | 0.38 |
|  | AA | 4 | 2 | 50.0 | 3.54 | (0.54 - 23.2) | 0.19 |
|  | CA +AA | 17 | 8 | 47.1 | 2.36 | (0.60 - 9.26) | 0.22 |
| 496 patients from Spain “WITHOUT ONLY IFN” ^b^ | | | | | | | |
| rs10964862 | **genotype** | **cases** | **n** | **%** | **HR*** | **CI*** | **P*** |
| OS | CC | 205 | 9 | 4.4 | 1.00 | (referent) | - |
|  | CA | 239 | 13 | 5.4 | 1.21 | (0.52 - 2.84) | 0.66 |
|  | AA | 47 | 5 | 10.6 | 2.41 | (0.80 - 7.20) | 0.12 |
|  | CA +AA | 286 | 18 | 6.3 | 1.41 | (0.63 - 3.14) | 0.41 |
| DFP | CC | 205 | 16 | 7.8 | 1.00 | (referent) | - |
|  | CA | 239 | 26 | 10.9 | 1.30 | (0.69 - 2.46) | 0.41 |
|  | AA | 47 | 8 | 17.0 | 2.52 | (1.07 - 5.90) | **0.03** |
|  | CA +AA | 286 | 34 | 11.9 | 1.48 | (0.81 - 2.71) | 0.20 |
| MD | CC | 18 | 12 | 66.7 | 1.00 | (referent) | - |
|  | CA | 31 | 14 | 45.2 | 0.58 | (0.24 - 1.40) | 0.23 |
|  | AA | 8 | 5 | 62.5 | 0.59 | (0.19 - 1.81) | 0.36 |
|  | CA +AA | 39 | 19 | 48.7 | 0.59 | (0.25 - 1.34) | 0.21 |

**^a^** only IFN as therapy

**^b^** no treatment

n number of deaths for OS and MD analysis or number of metastasis for DFP analysis

**^§^** adjusted for age, gender, Breslow thickness and treatment as time-dependent variable

*adjusted for age, gender and Breslow thickness

HR, Hazard Ratio; CI, Confidence Interval
