# Supplementary material for: Influence of Genetic Variants in Type I Interferon Genes on Melanoma Survival and Therapy
Source: PLoS One. 2012 Nov 27;7(11):e50692. doi: 10.1371/journal.pone.0050692 (PMC3507747; doi:10.1371/journal.pone.0050692)
Supplement: Table S11 — Variation rs597408 for the OS, DFS and MD analysis for the patients from Germany and Spain adjusted for the covariates age, gender and Breslow thickness. (DOCX) [file pone.0050692.s011.docx]

**Table S11. Variation rs597408 for the OS, DFS and MD analysis for the patients from Germany and Spain adjusted for the covariates age, gender and Breslow thickness**

| rs597408 | genotype | cases | n | % | HR | CI | P |
| --- | --- | --- | --- | --- | --- | --- | --- |
| OS GERMAN | AA | 466 | 101 | 21.7 | 1.00 | (referent) | - |
|  | AG | 50 | 9 | 18 | 0.89 | (0.45 - 1.77) | 0.75 |
|  | GG | 14 | 7 | 50 | 2.75 | (1.26 - 6.00) | **0.01** |
|  | AG +GG | 64 | 16 | 25 | 1.27 | (0.75 - 2.15) | 0.38 |
| OS SPANISH | AA | 580 | 36 | 6.2 | 1.00 | (referent) | - |
|  | AG | 45 | 4 | 8.9 | 2.08 | (0.73 - 5.92) | 0.17 |
|  | GG | 3 | - | - | - | - | - |
|  | AG +GG | 48 | 4 | 8.3 | 1.97 | (0.69 - 5.62) | 0.20 |
| DFS GERMAN | AA | 466 | 169 | 36.3 | 1.00 | (referent) | - |
|  | AG | 50 | 17 | 34 | 0.95 | (0.57 - 1.56) | 0.83 |
|  | GG | 14 | 8 | 57.1 | 2.16 | (1.05 - 4.43) | **0.04** |
|  | AG +GG | 64 | 25 | 39.1 | 1.16 | (0.77 - 1.78) | 0.50 |
| DFS SPANISH | AA | 580 | 69 | 11.9 | 1.00 | (referent) | - |
|  | AG | 45 | 7 | 15.6 | 1.77 | (0.81 - 3.89) | 0.16 |
|  | GG | 3 | 1 | 33.3 | 3.57 | (0.49 - 26.0) | 0.21 |
|  | AG +GG | 48 | 8 | 16.7 | 1.89 | (0.90 - 3.96) | 0.09 |
| MD GERMAN | AA | 185 | 115 | 62.2 | 1.00 | (referent) | - |
|  | AG | 21 | 10 | 47.6 | 0.87 | (0.46 - 1.67) | 0.68 |
|  | GG | 8 | 7 | 87.5 | 1.79 | (0.82 - 3.93) | 0.15 |
|  | AG +GG | 29 | 17 | 58.6 | 1.1 | (0.66 - 1.84) | 0.71 |
| MD SPANISH | AA | 78 | 38 | 48.7 | 1.00 | (referent) | - |
|  | AG | 8 | 5 | 62.5 | 0.97 | (0.38 - 2.51) | 0.95 |
|  | GG | 1 | - | - | - | - | - |
|  | AG +GG | 9 | 5 | 55.6 | 0.94 | (0.37 - 2.43) | 0.91 |

n, number of deaths for OS and MD analysis or number of metastases for DFS analysis

OS, overall survival; DFS, disease free progression; MD, metastasis to death

HR, Hazard Ratio; CI, Confidence Interval
