# Supplementary material for: Influence of Genetic Variants in Type I Interferon Genes on Melanoma Survival and Therapy
Source: PLoS One. 2012 Nov 27;7(11):e50692. doi: 10.1371/journal.pone.0050692 (PMC3507747; doi:10.1371/journal.pone.0050692)
Supplement: Table S12 — Estimated 10 years OS, DFS and MD survival analysis for the group of patients from Germany “with only IFN” ( Figure 2 D) and “without only IFN” ( Figure 2 E) for the SNP rs597408. (DOCX) [file pone.0050692.s012.docx]

**Table S12. Estimated 10 years OS, DFP and MD survival analysis for the group of patients from Germany “with only IFN” (Figure 2 D) and “without only IFN” (Figure 2 E) for the SNP rs597408**

| 84 patients from Germany “WITH ONLY IFN” ^a^ | | | | | | | |
| --- | --- | --- | --- | --- | --- | --- | --- |
| rs597408 | **genotype** | **cases** | **deaths** | **%** | **HR^§^** | **CI^§^** | **P^§^** |
| OS | AA | 72 | 13 | 18.1 | 1.00 | (referent) | - |
|  | AG | 10 | - | - | - | - | - |
|  | GG | - | - | - | - | - | - |
|  | AG +GG | 10 | - | - | - | - | - |
| DFP | AA | 72 | 29 | 40.3 | 1.00 | (referent) | - |
|  | AG | 10 | 6 | 60.0 | 1.47 | (0.58 - 3.74) | 0.41 |
|  | GG | - | - | - | - | - | - |
|  | AG +GG | 10 | 6 | 60.0 | 1.47 | (0.58 - 3.74) | 0.41 |
| MD | AA | 32 | 13 | 40.6 | 1.00 | (referent) | - |
|  | AG | 6 | - | - | - | - | - |
|  | GG | ­- | - | - | - | - | - |
|  | AG +GG | 6 | - | - | - | - | - |
| 457 patients from Germany “WITHOUT ONLY IFN” ^b^ | | | | | | | |
| rs597408 | **genotype** | **cases** | **deaths** | **%** | **HR*** | **CI*** | **P*** |
| OS | AA | 393 | 88 | 22.4 | 1.00 | (referent) | - |
|  | AG | 40 | 9 | 22.5 | 1.10 | (0.55 - 2.18) | 0.80 |
|  | GG | 14 | 7 | 50.0 | 2.63 | (1.20 - 5.76) | **0.02** |
|  | AG +GG | 54 | 16 | 29.6 | 1.47 | (0.86 - 2.51) | 0.16 |
| DFP | AA | 393 | 139 | 35.4 | 1.00 | (referent) | - |
|  | AG | 40 | 11 | 27.5 | 0.80 | (0.43 - 1.48) | 0.48 |
|  | GG | 14 | 8 | 57.1 | 2.09 | (1.01 - 4.29) | **0.05** |
|  | AG +GG | 54 | 19 | 35.2 | 1.08 | (0.67 - 1.75) | 0.75 |
| MD | AA | 152 | 102 | 67.1 | 1.00 | (referent) | - |
|  | AG | 15 | 10 | 66.7 | 1.20 | (0.62 - 2.33) | 0.58 |
|  | GG | 8 | 7 | 87.5 | 1.75 | (0.79 - 3.86) | 0.17 |
|  | AG +GG | 23 | 17 | 73.9 | 1.38 | (0.81 - 2.34) | 0.23 |

**^a^** only IFN as treatment

**^b^** no therapy or different kinds of therapies combined or not with IFN

n number of deaths for OS and MD analysis or number of metastasis for DFP analysis

**^§^** adjusted for age, gender, Breslow thickness and treatment as time-dependent variable

*adjusted for age, gender and Breslow thickness

HR, Hazard Ratio; CI, Confidence Interval
